# Analyses of energy metabolism and stress defence provide insights into *Campylobacter concisus* growth and pathogenicity

**DOI:** 10.1186/s13099-020-00349-6

**Published:** 2020-03-05

**Authors:** Melissa Yeow, Fang Liu, Rena Ma, Timothy J. Williams, Stephen M. Riordan, Li Zhang

**Affiliations:** 1 School of Biotechnology and Biomolecular Sciences, University of New South Wales, Kensington, Sydney, 2052 Australia; 2 Gastrointestinal and Liver Unit, Prince of Wales Hospital, University of New South Wales, Sydney, Australia

**Keywords:** *Campylobacter concisus*, *Campylobacter jejuni*, *Campylobacter*, Hydrogen, Energy, Metabolism, Respiration

## Abstract

*Campylobacter concisus* is an emerging enteric pathogen that is associated with inflammatory bowel disease. Previous studies demonstrated that *C. concisus* is non-saccharolytic and hydrogen gas (H_2_) is a critical factor for *C. concisus* growth. In order to understand the molecular basis of the non-saccharolytic and H_2_-dependent nature of *C. concisus* growth, in this study we examined the pathways involving energy metabolism and oxidative stress defence in *C. concisus*. Bioinformatic analysis of *C. concisus* genomes in comparison with the well-studied enteric pathogen *Campylobacter jejuni* was performed. This study found that *C. concisus* lacks a number of key enzymes in glycolysis, including glucokinase and phosphofructokinase, and the oxidative pentose phosphate pathway. *C. concisus* has an incomplete tricarboxylic acid cycle, with no identifiable succinyl-CoA synthase or fumarate hydratase. *C. concisus* was inferred to use fewer amino acids and have fewer candidate substrates as electron donors and acceptors compared to *C. jejuni*. The addition of DMSO or fumarate to media resulted in significantly increased growth of *C. concisus* in the presence of H_2_ as an electron donor, demonstrating that both can be used as electron acceptors. Catalase, an essential enzyme for oxidative stress defence in *C. jejuni*, and various nitrosative stress enzymes, were not found in the *C. concisus* genome. Overall, *C. concisus* is inferred to have a non-saccharolytic metabolism in which H_2_ is central to energy conservation, and a narrow selection of carboxylic acids and amino acids can be utilised as organic substrates. In conclusion, this study provides a molecular basis for the non-saccharolytic and hydrogen-dependent nature of *C. concisus* energy metabolism pathways, which provides insights into the growth requirements and pathogenicity of this species.

## Introduction

*Campylobacter* species are fastidious Gram-negative, curved rod-shaped bacteria which require microaerobic to anaerobic conditions for growth [1]. Currently, the genus *Campylobacter* contains 40 species and subspecies [2]. While most *Campylobacter* species reside in the gastrointestinal tract of various animals as commensal bacterial species, some use humans as their natural host [2].

*Campylobacter jejuni* colonises the avian gut commensally, but it is a human pathogen causing gastroenteritis in both developing and developed countries [3–5], due to consumption of undercooked or contaminated chicken and other meat products [6–8]. *C. jejuni* contains two subspecies, *C. jejuni* subsp. *jejuni* and *C. jejuni* subsp. *doylei* [9]. Most of the cases of campylobacteriosis are caused by *C. jejuni* subsp. *jejuni* [10].

*Campylobacter concisus* is a human hosted *Campylobacter* species that colonizes the oral cavity of healthy individuals [11]. It is also an emerging enteric pathogen that has been associated with the development of inflammatory bowel disease (IBD) and other gastrointestinal diseases [12–19].

*Campylobacter* species have been historically considered to be non-saccharolytic, given that they have an incomplete Embden–Meyerhof–Parnas (EMP) glycolytic pathway that lacks hexose catabolism enzymes such as glucokinase and phosphofructokinase [20]. However, subsequent studies have amended this view. *C. jejuni* subsp. *doylei* 269.97 encodes a complete Entner–Doudoroff (ED) pathway, putatively acquired from *Helicobacter*, which suggests the potential to catabolise glucose [21]. The ED pathway has also recently been found in certain strains of the zoonotic enteric pathogen *Campylobacter coli* [22]. In addition, *C. jejuni* subsp. *jejuni* strains that have the genomic island (*cj0480*–*cj0490*) were demonstrated to have the ability to utilise fucose for growth, which is catabolized to pyruvate and lactate [23, 24]. The lower portion of the EMP is encoded in *C. jejuni*, and inferred to be involved in gluconeogenesis, which is dependent on anaplerotic enzymes to link it to the tricarboxylic acid (TCA) cycle [25].

As most strains of *C. jejuni* cannot utilise sugars [24, 26, 27], *C. jejuni* mainly relies on amino acids and C4-dicarboxylates as a primary energy source [28]. *C. jejuni* is known to preferentially use the amino acids aspartate, serine, glutamate, asparagine and proline [29–32], as well as the C4-dicarboxylates fumarate, succinate and malate [30]. Use of amino acids and C4-dicarboxylates for energy metabolism are interconnected as amino acids can be converted into C4-dicarboxylates that can enter the TCA cycle or be used in the electron transport chain for energy metabolism. Additionally, the C4-dicarboxylate transporters DcuA and DcuB found in *C. jejuni* are known to transport both fumarate and the dicarboxylic amino acid aspartate [28, 33].

*C. jejuni* has a highly branched electron transport chain, with a range of electron transport routes available, and the ability to use diverse substrates as electron donors [25, 34]. This includes organic acids such as formate [35, 36], gluconate [37, 38], lactate [39], and in particular the TCA cycle intermediates pyruvate [40], 2-oxoglutarate [41], succinate [42], fumarate [42] and malate [36]. Hydrogen gas (H_2_) [35, 43] and sulphite [44] can also be used as electron donors. In addition to oxygen-dependent respiration [45], a variety of alternative electron acceptors can be used by *C. jejuni* including fumarate [42, 46], nitrate [47], nitrite [48], trimethylamine N-oxide (TMAO), dimethyl sulphoxide (DMSO) [49], and tetrathionate [50].

The molecular basis of the non-saccharolytic and hydrogen-dependent nature of *C. concisus* has not been previously made clear, because few studies have examined the genes and pathways involved in respiration and energy metabolism in *C. concisus* [51, 52]. Recently, more than 200 *C. concisus* genomes including three complete genomes have become available in public databases, providing the opportunity for us to investigate these pathways using bioinformatics analysis. This study investigates the interlinked pathways of central carbon metabolism, the electron transport chain, amino acid use, and oxidative stress defence mechanisms as previous studies on these pathways in *C. jejuni* have shown their importance for growth, motility, survival of the host immune response, and host colonization [25, 34, 42, 43, 53, 54].

## Results

### *C. concisus* has an incomplete glycolytic pathway and an incomplete pentose phosphate (PP) pathway

**Fig. 1:**
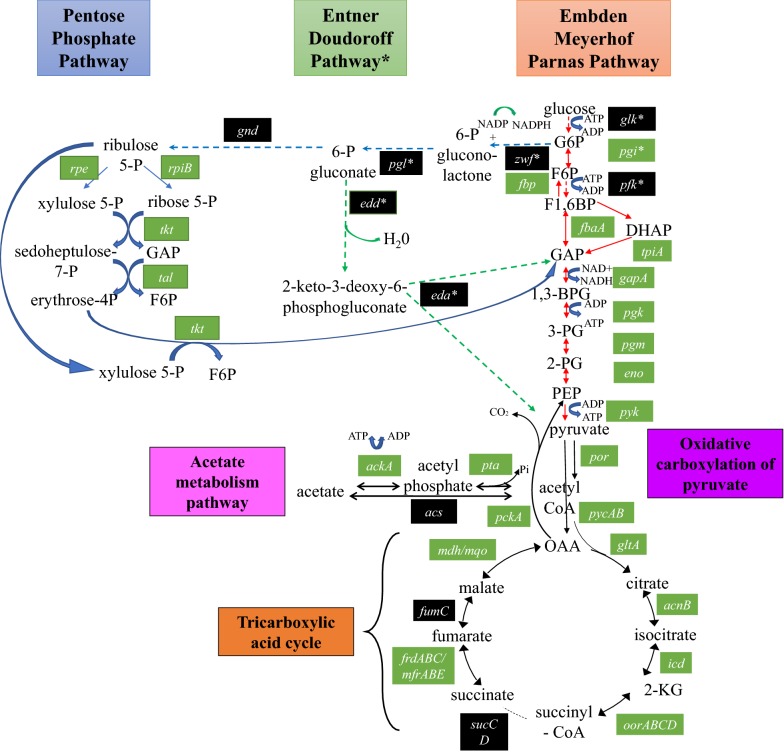
Enzymes and their genes of central carbon metabolism pathways identified in *C. concisus.* The figure illustrates the pathways of the central carbon metabolisms in *C. concisus* compared with *C. jejuni*, drawn with reference to the Kyoto Encyclopedia of Genes and Genomes [73]. The enzymes in this figure and their functions are found in Additional file 1: Table S1, Additional file 3: Table S3 and Additional file 4: Table S4. *G6P* glucose-6-phosphate, *F6P* fructose-6-phosphate, *F1*,*6BP* fructose 1,6-bisphosphate, *GAP* glyceraldehyde-3-phosphate, *1*,*3-BPG* 1,3-bisphosphoglycerate, *3PG* 3-phosphoglycerate, *2PG* 2-phosphoglycerate, *PEP* phosphoenolpyruvate, *ribulose 5P* ribulose 5-phosphate, *ribose 5P* ribose 5-phosphate, *xylulose 5P* xylulose 5-phosphate, *sedoheptulose 7P* sedoheptulose 7-phosphate, *erythrose-4P* erythrose 4-phosphate, *6P gluconate* 6-phosphogluconate, *OAA* oxaloacetate, *2-KG* 2-oxoglutarate. Genes and proteins in green boxes were found in *C. concisus* and those in black boxes were not identified in *C. concisus*. The query genes and proteins used in identification of similar genes and proteins in *C. concisus* were from strains in *C. jejuni* subsp. *jejuni* NCTC 11168 and *C. jejuni* subsp. *doylei* 269.97 (*)

**Fig. 2:**
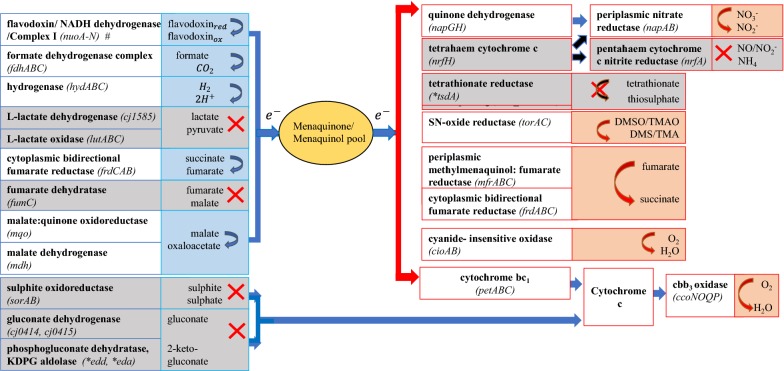
Respiratory enzymes and their genes identified in *C. concisus.* Respiratory enzymes and their pathways are shown in this figure and the genes encoding the enzymes are in italic. The enzymes in this figure and their functions are in Additional file 2: Table S2, Additional file 5: Table S5 and Additional file 11: Table S11. Genes encoding for respiratory enzymes are shown in white boxes. Electron donors are shown in blue and electron acceptors are shown in red. Genes not identified in *C. concisus* are shaded gray and marked with a red cross. The ED pathway genes were referenced from *C. jejuni* subsp. *doylei* 269.97 and the tetrathionate reductase genes *tsdA C8J_0815* and *C8J_0040* were referenced from *C. jejuni* strain 81116

The EMP pathway of *C. concisus* is incomplete, lacking the genes for glucokinase (*glk*) and phosphofructokinase (*pfk*) (Fig. 1 and Additional file 1: Table S1), but possessing the gene for phosphoglucose isomerase (*pgi*) and all other genes, including those required for gluconeogenesis. A complete gluconeogenesis pathway is present in *C. concisus*: pyruvate carboxylase (*pycAB*), phosphoenolpyruvate carboxykinase (*pckA*), and fructose 1,6-bisphosphatase (*fbp*) have been identified (Fig. 1 and Additional file 1: Table S1). The PP pathway in *C. concisus* is incomplete; genes pertaining to the oxidative phase were not identified: glucose 6-phosphate dehydrogenase (*gdh*) and 6-phosphogluconate dehydrogenase (*zwf*) (Fig. 1 and Additional file 1: Table S1). None of the genes of the ED pathway were identified in *C. concisus* (Figs. 1, 2, Additional file 1: Table S1 and Additional file 2: Table S2). Thus, *C. concisus* is unable to catabolise glucose to pyruvate through the EMP, PP or ED pathways. *C. concisus* lacks homologs of *C. jejuni* genes implicated in the catabolism of fucose (*cj0481*, *cj0484*, *cj0485*, *cj0486*, *cj0487*). Genes for the conversion of pyruvate to acetate via acetyl-CoA and acetylphosphate were identified in *C. concisus*: pyruvate-flavodoxin oxidoreductase (*por*), acetate kinase (*ackA*), and phosphate acetyltransferase (*pta*). (Additional file 1: Table S1). A H_2_-generating [NiFe] hydrogenase (Group 4a) was identified in the genome, for the disposal of reductant as H_2_.

### *C. concisus* has an incomplete TCA cycle

Most of the genes in the TCA cycle were identified in *C. concisus* except for succinyl-CoA synthetase (*sucCD*) and fumarate hydratase (*fumC*). A C4-dicarboxylate transporter (*dctA*) is encoded for the uptake of the TCA cycle intermediate succinate. Other C4-dicarboxylate transporters (*dcuA*, *dcuB*) are encoded for the uptake of the TCA cycle intermediate fumarate and aspartate. The genes required for the glyoxylate bypass were not identified in *C. concisus*: isocitrate lyase (*aceA*) and malate synthase (*aceB*) (Fig. 1, Additional file 1: Table S1, Additional file 3: Table S3 and Additional file 4: Table S4).

### *C. concisus* may be able to use fewer electron donors than *C. jejuni*

Genes for lactate oxidase (*lutCBA*) and lactate dehydrogenase (*ldh*) could not be identified in *C. concisus*, thus precluding use of the two pathways for use of lactate as an electron donor (Fig. 2 and Additional file 2: Table S2). *C. concisus* also lacks identifiable genes for gluconate dehydrogenase (*gndAB*). Given the absence of the ED pathway (see above) *C. concisus* is unable to catabolise gluconate to pyruvate (Fig. 2 and Additional file 1: Table S1 and Additional file 2: Table S2).

However, genes encoding enzymes for the oxidation of other electron donors were identified in *C. concisus*, such as malate:quinone oxidoreductase (*mqo*), pyruvate:flavodoxin oxidoreductase (*por)*, 2-oxoglutarate:acceptor oxidoreductase (*oorABCD*), formate dehydrogenase (*fdhABC*), succinate dehydrogenase (*frdABC*), and H_2_-uptake [NiFe] hydrogenase (*hydABCD*) (Group 1b) (Fig. 2 and Additional file 2: Table S2).

### *C. concisus* may be able to use fewer electron acceptors than *C. jejuni*

There are two pathways in *C. concisus* for use of oxygen as an electron acceptor, via cytochrome C oxidase (encoded by *ccoNOQP*), or cyanide-insensitive quinol oxidase (*cioAB*). Genes encoding nitrite reductase (*nrfAH*) were not identified in *C. concisus*, suggesting an inability to use nitrite as an electron acceptor. Also, homologs for the *C. jejuni* tetrathionate reductase *tsdA* (*C8J_0815*) and a *tsdA* paralog (*C8J*_*0040*) were not found in *C. concisus* (Fig. 2).

Genes encoding the periplasmic nitrate reductase (*napABGH*) which allows nitrate to be used as an electron acceptor, were found in *C. concisus.* Genes encoding the periplasmic methylmenaquinol fumarate reductase (*mfrABE*) as well as a dual-functioning cytoplasmic fumarate reductase (*frdABC*) were found in *C. concisus* (Fig. 2 and Additional file 5: Table S5). Methylmenaquinol fumarate reductase (Mfr) and fumarate reductase (Frd) allow use of fumarate as an electron acceptor, reducing fumarate to succinate using electrons from the menaquinone pool. *C. concisus* encodes an anaerobic C_4_-dicarboxylate membrane transporter (*dcuB*) for succinate efflux. TMAO/DMSO reductase (*torAC*) for use of SN-oxides as electron acceptors were also found in *C. concisus* (Fig. 2 and Additional file 5: Table S5).

### *C. concisus* may be able to use fewer amino acids than *C. jejuni*

**Fig. 3:**
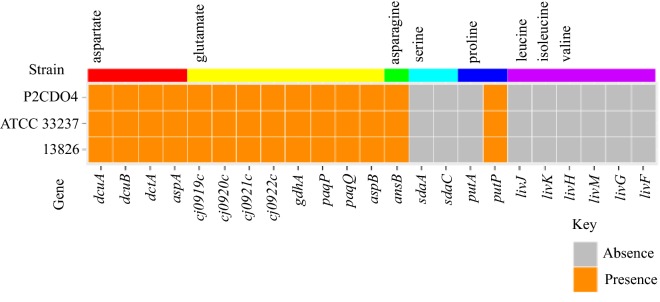
Genes for enzymes of amino acid use pathways identified in *C. concisus.* The genes for the enzymes of amino acid use pathways identified in *C. concisus* are shown in this figure. The corresponding amino acid used by the enzymes is shown by the colour coded bar above. The enzymes in this figure and their functions are in Additional file 6: Table S6 and Additional file 7: Table S7

Genes for the serine transporter (*sdaC*), serine dehydratase (*sdaA*) and proline dehydratase (*putA*) were not found in *C. concisus*, though the proline symporter (*putP*) was identified. Additionally, branched chain amino acids do not appear to be taken up by *C. concisus*, due to lack of the LIV transporter system (*livJKHMGF*). The pathogenesis associated glutamine ABC transporter permease (*paqP*) and ATPase (*paqQ*) as well as the ABC transporter system encoded by the *peb* locus which transports glutamate and aspartate were identified in *C. concisus*. Glutamate dehydrogenase (*ghdA*) and aspartate:glutamate transaminase (*aspB*) were also encoded, which suggests that *C. concisus* can convert glutamate to 2-oxoglutarate and ammonia, or generate aspartate and 2-oxoglutarate from glutamate and oxaloacetate via transamination. Aspartate ammonia lyase (*aspA*) was also encoded, for conversion of aspartate to fumarate and ammonia. Periplasmic asparaginase (*ansB*) was also found, allowing use of asparagine as a source of aspartate (Fig. 3, Additional file 6: Table S6 and Additional file 7: Table S7).

### *C. concisus* has fewer enzymes to deal with oxidative and nitrosative stress than *C. jejuni*

**Fig. 4:**
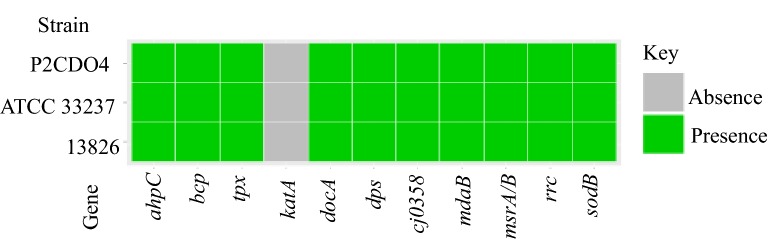
Genes for enzymes of oxidative stress defence pathways identified in *C. concisus.* The genes for the enzymes of oxidative stress defence pathways identified in *C. concisus* are shown in this figure. The enzymes in this figure and their functions are in Additional file 8: Table S8 and Additional file 9: Table S9

The *katA* gene, which encodes catalase that detoxifies hydrogen peroxide, was not found in *C. concisus*. Ten other genes that are involved in dealing with oxidative stress in *C. jejuni* were found in *C. concisus* (Fig. 4). The three genes that encode enzymes to deal with nitrosative stress in *C. jejuni* (*cgb*, *ctb*, *nrfa*), were not found in *C. concisus*.

### Without the presence of H_2_, sodium fumarate did not increase *C. concisus* growth

**Fig. 5:**
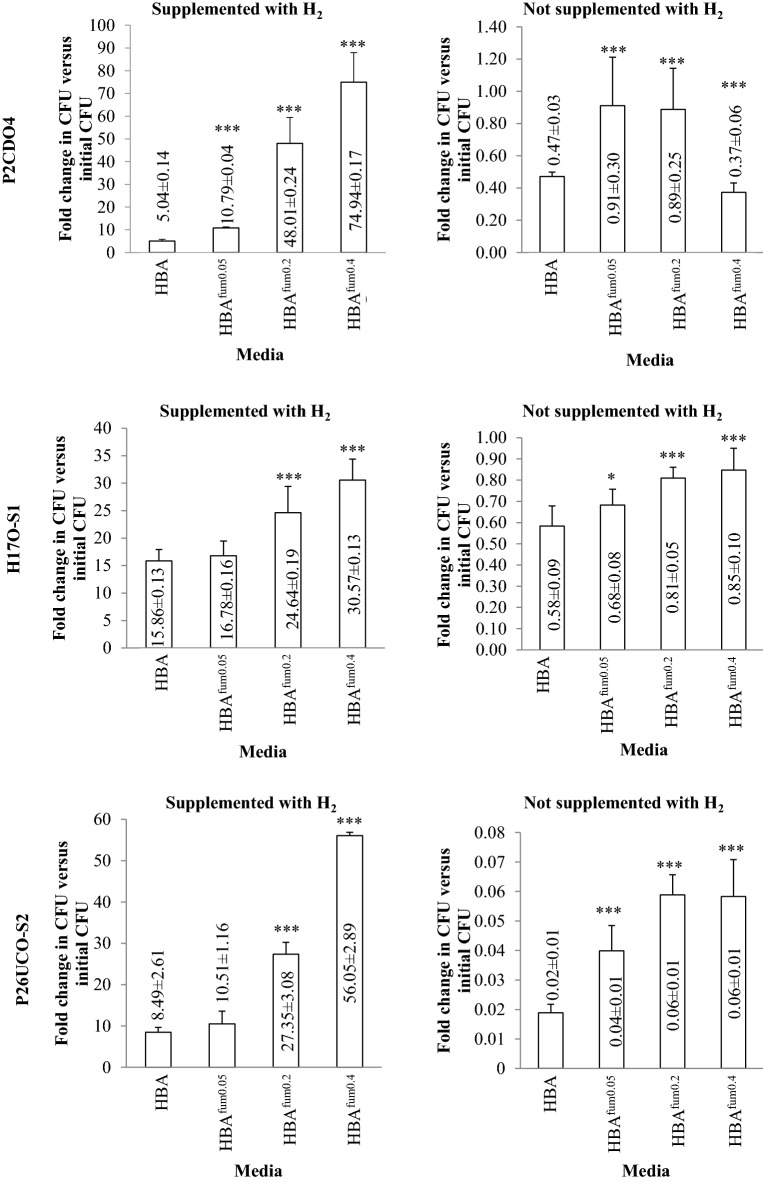
The effects of sodium fumarate on *C. concisus* growth under anaerobic conditions with and without H_2_ gas. *C. concisus* strains P2CDO4, H17O-S1, and P26UCO-S2 were cultured on two sets of HBA, HBA^fum0.05^, HBA^fum0.2^ and HBA^fum0.4^. One set was incubated in anaerobic conditions with hydrogen and the other was incubated without hydrogen. Both the starting number of bacteria and total after incubation was enumerated for all strains. Fold changes were calculated relative to the starting number of bacteria from quadruplicate counts. In anaerobic conditions with hydrogen, all strains showed increased growth, with the greatest increase on HBA^fum0.4^. In anaerobic conditions without hydrogen, all strains had a CFU lower than the initial inoculation CFU. *Indicates statistical significance compared to CFU on HBA in the same condition. *** *P* < 0.001

The effects of sodium fumarate on *C. concisus* growth under anaerobic conditions with and without the presence of H_2_ were examined. Under anaerobic conditions with H_2_, all three strains cultured on horse blood agar (HBA) plates and HBA plates supplemented with 0.05, 0.2 and 0.4% (w/v) sodium fumarate showed an increase in colony forming unit (CFU) compared to the initial inoculum, with fold changes greater than one (Fig. 5). Compared to the same strains grown on HBA plates, all three strains grown on HBA plates supplemented with 0.2 and 0.4% sodium fumarate had a significantly higher CFU (*P* < 0.05). Strain P2CDO4 also showed a significantly higher growth on HBA plates supplemented with 0.05% sodium fumarate as compared to the same strain grown on HBA plates (*P* < 0.05, Fig. 5).

In conditions without H_2_, all three *C. concisus* strains did not grow; their CFU fold changes cultured on HBA plates and HBA plates supplemented with different concentrations of sodium fumarate were all below one as compared to the initial inoculum CFU. Although the decrease in *C. concisus* CFU on HBA plates containing 0.05–0.04% was less than that in HBA plates without sodium fumarate, the overall results show that sodium fumarate did not increase *C. concisus* growth without the presence of H_2_ (Fig. 5).

### DMSO increased the growth of *C. concisus*

**Fig. 6:**
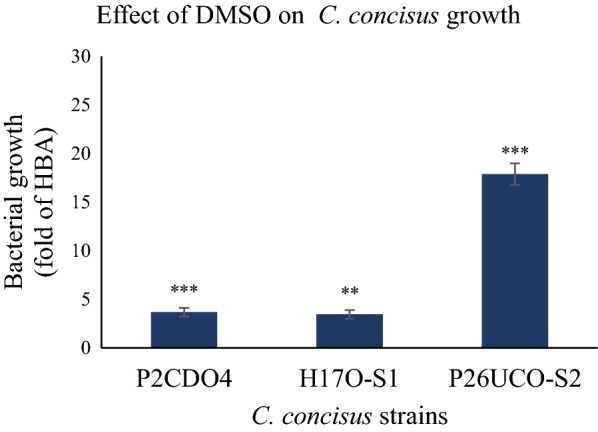
The effects of dimethyl sulphoxide (DMSO) on *C. concisus* growth. *C. concisus* strains P2CDO4, H17O-S1, and P26UCO-S2 were cultured on HBA plates or HBA plates supplemented with 0.2% DMSO. DMSO enhanced the growth of all three *C. concisus* strains being tested. The levels of growth of *C. concisus* strains P2CDO4, H17O-S1, and P26UCO-S2 on HBA plates containing DMSO were 4 ± 0.4, 3 ± 0.4, and 18 ± 1.1 folds of those on HBA plates without DMSO, in which the differences were statistically significant. *Indicates statistical significance. **P < 0.01. ***P < 0.001

In order to examine the effects of DMSO on *C. concisus* growth, *C. concisus* strains were cultured on HBA plates containing 0.2% of DMSO (HBA^DMSO^). DMSO significantly enhanced the growth of all three *C. concisus* strains being tested (Fig. 6). The levels of growth of *C. concisus* strains P2CDO4, H17O-S1, and P26UCO-S2 on HBA^DMSO^ plates were 4 ± 0.4, 3 ± 0.4 and 18 ± 1.1 fold of those on HBA plates respectively, in which the increases were statistically significant (*P* < 0.001, *P* < 0.01, and *P* < 0.001 respectively).

## Discussion

In this study, we examined the pathways of energy metabolism and stress defence in *C. concisus* by analysis of the presence of relevant genes in *C. concisus* genomes. We also examined whether sodium fumarate increased *C. concisus* growth without the presence of H_2_ and whether DMSO affects the growth of *C. concisus*.

We found that *C. concisus* does not have complete metabolic pathways for the utilization of glucose [55]. The lack of *glk* and *pfk* genes indicates that *C. concisus* is unable to convert glucose to fructose-1,6-bisphosphate using the EMP pathway. The lack of *gdh* and *zwf* shows that *C. concisus* is unable to metabolise glucose via the PP pathway (Fig. 1). Furthermore, *C. concisus* does not have genes for the ED pathway or for fucose catabolism, unlike as documented in certain *C. jejuni* strains (Fig. 1 and Additional file 1: Table S1 and Additional file 5: Table S5) [38]. Collectively, these findings support the previous characterization of *C. concisus* as non-saccharolytic [1].

We found that *C. concisus* has an incomplete TCA cycle (Fig. 2, Additional file 1: Table S1). Although *C. concisus* encodes most of the enzymes in the TCA cycle, it lacks both *fumC* and *sucCD*; the former is required for the production of fumarate from malate, the latter is necessary for generation of succinate from succinyl-CoA. It has previously been reported that succinate is an end product of *C. concisus* growth, generated from fumarate reduction [55]. The two enzymes Frd or Mfr are available in *C. concisus* to reduce fumarate (Fig. 2). During oxygen-independent respiration, fumarate reduction in *C. jejuni* generates succinate that is secreted during growth [28], and a DcuB transporter is encoded for succinate efflux.

*C. concisus* has the capacity to produce most TCA cycle intermediates, which donate reducing equivalents to the respiratory chain for energy metabolism (Fig. 2, Additional file 1: Table S1). Although a fumarate hydratase was initially annotated in *C. concisus*, the genes are more likely to encode tartrate dehydratase based on sequence identity [56]. *C. concisus* encodes aspartate ammonia-lyase (*aspA*); aspartate is a likely source of fumarate in *C. concisus*, in addition to fumarate directly sourced from the host diet [57]. The genome encodes transporters for the uptake of both aspartate and fumarate. The inability to use proline and serine by *C. concisus* (Fig. 3, Additional file 6: Table S6 and Additional file 7: Table S7) is interesting as proline utilization is considered important for *C. jejuni* intestinal colonization of mice [58], and serine utilization is critical for *C. jejuni* host colonization in chicks and mice [58, 59].

The genes necessary for pyruvate conversion to acetate (*por*, *pta*, *ackA*) were found in *C. concisus*; thus we infer the potential to generate ATP via substrate-level phosphorylation. It has been found previously that *C. concisus* generated acetate, although strain variation was noted by that study which detected acetate in three out of six *C. concisus* strains they examined [55].

Our findings also provide an explanation at the genomic level for *C. concisus* growth requirements. *C. jejuni* has genes encoding enzymes for all reactions in the TCA cycle; thus it is able to generate reducing equivalents for ATP production via the electron transport chain. In contrast, *C. concisus* is unable to use succinyl-CoA to generate succinate, fumarate, malate, FADH_2_ and NADH molecules in the TCA cycle due to the lack of *sucCD* genes and cannot generate fumarate from malate due to lack of *fumC* (Fig. 1).

However, *C. concisus* has genes encoding enzymes for utilizing the amino acids glutamate, aspartate and asparagine (Fig. 3), as well as several electron donors, such as H_2_, formate, succinate and malate (Fig. 2). Given this, supplementation of these amino acids and electron donors may potentially increase the growth of *C. concisus* and the effects of some of these substances on *C. concisus* growth were examined previously and in this study.

H_2_ gas has been demonstrated to be a highly effective electron donor for *C. concisus* growth [51, 60]. It has been previously demonstrated that *C. concisus* had very low growth under anaerobic conditions and no growth under microaerobic conditions without H_2_ [42]. In the presence of H_2_, *C. concisus* was able to grow under microaerobic conditions and the growth under anaerobic conditions greatly increased [42]. *C. concisus* has both a H_2_-uptake hydrogenase (Hyd) and a H_2_-generating hydrogenase (Hyf) [51]. Site-directed mutagenesis demonstrated the critical role of H_2_ oxidation for *C. concisus* growth, with *C. concisus* additionally found to exhibit extremely high H_2_-uptake hydrogenase activity [51]. Although H_2_ evolution has been detected for *C. concisus* [55], the level of H_2_ produced by *C. concisus* produced only poor growth of *C. concisus* [60]. Thus, uptake of exogenous H_2_ generated by other bacterial species is a critical factor affecting the growth of *C. concisus* in the human gastrointestinal tract.

Fumarate can be used as an electron acceptor, with genes encoding a periplasmic Mfr and a bifunctional cytoplasmic Frd present in *C. concisus* (Fig. 2). However, as with the periplasmic nitrate reductase (Nap), Frd cannot contribute to generation of the membrane potential, and functions solely as an electron sink [33]. Sodium fumarate has been previously demonstrated to greatly increase the growth of *C. concisus* in the presence of H_2_ [61], which in combination with our study, showed that fumarate is an effective electron acceptor in *C. concisus*. This is further supported by proteomic data which show that fumarate reductase proteins (WP_012001794.1, WP_012001792.1, WP_012001793.1) had increased abundance in response to addition of fumarate [61].

Formate is also a potential electron donor in *C. concisus*, which encodes the formate dehydrogenase complex (*fdhABC*) (Fig. 2). Further, formate oxidation might be coupled to a H_2_-generating hydrogenase in a formate hydrogen lyase complex as in *Escherichia coli* [62]. However, previous studies found that supplementation of media with formate did not support *C. concisus* growth. *C. concisus* growth was inhibited by 0.2% formate in anaerobic conditions with 5% hydrogen [61]; growth did not occur in 20 mM formate supplemented media under microaerobic conditions (5% oxygen) without hydrogen; and poor growth was observed in 20 mM formate supplemented media under anaerobic conditions without hydrogen [51]. Further investigation into the effect of different concentrations of formate on *C. concisus* is warranted to shed light on this paradoxical phenomenon.

This study found that *C. concisus* has genes encoding enzymes for using nitrate, DMSO/TMAO, fumarate, and O_2_ as electron acceptors (Fig. 2). As mentioned above, fumarate is an effective electron acceptor in *C. concisus* [61]. The good growth of *C. concisus* under anaerobic conditions in the presence of H_2_ suggests that nitrate present in media may be used as an electron acceptor. In this study, we showed that DMSO increased the growth of *C. concisus* in the presence of H_2_, showing that it can be used effectively as an electron acceptor in *C. concisus* (Fig. 6). While nitrite reductase genes *nrfCD* have been previously reported in *C. concisus* [51], it is unlikely to be functional due to lack of a gene for the catalytic subunit NrfA [48]. *C. concisus* also has the potential to use O_2_ as a terminal electron acceptor as it has the genes encoding a cyanide-insensitive oxidase (*cioAB*) and ubiquinol-cytochrome c oxidoreductase (*ccoNOQP*), also referred to as the cytochrome bc_1_ complex (Fig. 2). However, the abilities of *C. concisus* using O_2_ as an electron acceptor may have been limited due to its lack of catalase, which was found to be essential for hydrogen peroxide resistance in *C. jejuni* [53].

Catalase catalyzes the decomposition of hydrogen peroxide, a highly reactive product generated during using molecular oxygen as the final electron acceptor. *C. concisus* does not have *katA* (Fig. 4, Additional file 8: Table S8 and Additional file 9: Table S9) and was previously demonstrated to be catalase negative [55]. *C. concisus* has the gene encoding superoxide dismutase, which was found to be expressed in *C. concisus* when fumarate was added to media [61]. The overall ability of *C. concisus* in dealing with reactive oxygen stress appears lower compared to *C. jejuni*, which has both catalase and superoxide dismutase [53, 63]. This is especially likely as it has been shown that catalase is essential for *C. jejuni* in vitro hydrogen peroxide resistance, and intramacrophage survival via *katA* mutant experiments [53]. This may explain a previous observation that *C. concisus* grew better under anaerobic than microaerobic conditions in the presence of 5% H_2_ [60]. *C. concisus* has been demonstrated to grow better in microaerobic conditions than in anaerobic conditions when H_2_ concentration was raised to 20% [51]. This interesting observation that a higher level of H_2_ appeared to increase the ability of *C. concisus* to resist oxidative stress may be due to antioxidant effects of hydrogen via reduction of hydroxyl radicals and peroxynitrite [64].

*C. concisus* does not have any of the three known nitrosative stress defence enzymes used by *C. jejuni.* However, it was previously reported that *C. concisus* strain 13826 has a nitric oxide reductase NorZ and a nitrous oxide reductase NosZ [51], which are not present in *C. jejuni* [65]. This study found that these enzymes were present in all three fully sequenced strains of *C. concisus*. This may explain the increased growth of *C. concisus* strain ATCC 33237 in response to an increased concentration of nitric oxide donor sodium nitroprusside [66].

Whereas we found that *C. concisus* lacked the gene that encodes tetrathionate reductase in *C. jejuni* strain 81116, a tetrathionate reductase gene has been reported in *C. concisus* strain 13826 that is similar to the tetrathionate reductase (*ttr*) in *Salmonella typhimurium* [51]. The latter study also demonstrated a functional tetrathionate reduction pathway using site-directed mutagenesis, and found that addition of tetrathionate to growth media resulted in increased growth of *C. concisus* [51].

Findings from this study and previous studies suggest that the pathogenicity of *C. concisus* is not only determined by the virulence of individual *C. concisus* strains but also the microenvironment of the gastrointestinal tract of individual hosts particularly the availability of H_2_ for growth. As the composition of microbiota and diet are the two major factors influencing the production of H_2_ in the gastrointestinal tract, their impact on *C. concisus* enteric pathogenicity warrants future investigation.

## Conclusions

This study found that *C. concisus* lacks critical genes in the central carbon metabolism pathways and has an incomplete TCA cycle, with a missing succinyl-CoA synthetase (*sucCD*) and fumarate hydratase (*fumC*). This study showed that fumarate and DMSO are effective electron acceptors in *C. concisus* in the presence of H_2_ as an electron donor. *C. concisus* was also found to have fewer genes that encode enzymes for utilizing amino acids, electron donors and acceptors, as well as stress defence compared to *C. jejuni*, although this study cannot rule out the possibility that *C. concisus* may use other alternative pathways. In conclusion, this study provides a molecular basis for the non-saccharolytic and hydrogen-dependent nature of *C. concisus* via its energy metabolism and stress defence pathways, which provides insights into the growth requirements and pathogenicity of this species.

## Materials and methods

### Bioinformatic methods used to examine the presence of genes encoding enzymes in energy production and stress defence in *C. concisus*

The sequences of the reference genes from reference bacterial strains (see the section below) were obtained from the National Center for Biotechnology Information (NCBI) database. Proteins encoded by these genes were used as query sequences to identify similar proteins in the three *C. concisus* strains with fully sequenced genomes (13826, ATCC 33237 [67] and P2CDO4 [15]) using the BLASTp program [68]. Previously published criteria (more than 30% identity, E-values < 10^-10^ and bit scores of > 50) were used to determine presence of a gene [69]. Protein sequences that were identified as hydrogenases based on catalytic domains were classified further using the hydrogenase classifier HydDB [70].

The nucleotide sequences of the reference genes were also used as query genes to identify similar nucleotide sequences in the genomes of all 249 *C. concisus* strains in the public database including the three fully sequenced genomes using BLASTn as further verification of the data obtained from using the BLASTp comparison using the previously mentioned criteria [69]. The *C. concisus* strains used in the analysis of this study are in Additional file 10: Table S10.

### The reference genes used to identify similar genes and proteins in the *C. concisus* genomes

To examine the presence of genes encoding enzymes in the pathways of energy production and stress defence in *C. concisus*, a total of 206 reference genes and their encoded proteins from *E. coli* and *C. jejuni* were used as query genes and proteins to identify similar genes and proteins in *C. concisus* genomes using the methods described above.

Reference genes and their encoded proteins from *E. coli* strain K-12 MG1655 (NC_000913.3), *C. jejuni* subsp. *jejuni* strain NCTC 11168 (NC_002163.1) and *C. jejuni* subsp. *doylei* strain 269.79 (NC_009707.1) were used as the query genes and proteins. The choice of *E. coli* strain K-12 MG1655 as a reference strain was due to its well-studied metabolic pathways, in particular its glycolytic pathways [71]. *C. jejuni* strain NCTC 11168 (NC_002163.1) was used as a reference because it is a member of the *Campylobacter* genus with well-studied metabolic pathways [26, 72], except for the ED pathway, which was discovered in *C. jejuni* subsp. *doylei* 269.97 (NC_009707.1) and not found in most *C. jejuni* subsp. *jejuni* strains [38]. In addition, the tetrathionate reductase genes *tsdA* (*C8J_0815*) and the *tsdA* paralog (*C8J_0040*) were discovered in *C. jejuni* strain 81116 [50]. As such, *C. jejuni* strain 81116 was used as a reference for the tetrathionate reductase pathway.

The reference genes and encoding proteins that were used as query genes and proteins for identification of similar genes and enzymes in the central carbon metabolic pathways of *C. concisus* are listed in Additional file 3: Table S3 (*E. coli* strain K-12 reference genes) and Additional file 4: Table S4 (*C. jejuni* subsp. *jejuni* and *C. jejuni* subsp. *doylei* reference genes). The reference genes and proteins that were used as query genes or proteins for identification of similar respiratory chain enzymes in *jejuniC. concisus* are listed in Additional file 11: Table S11 (*C. jejuni* subsp. reference genes). The reference genes and encoded proteins from *C. jejuni* subsp. *jejuni* NCTC 11168, *C. jejuni* strain 81116 (*livJKHMGF*) and *E. coli* strain K-12 (*gdhA*) were used as query genes and proteins for identification of enzymes involved in amino acid use in are listed in Additional file 7: Table S7. The reference genes and encoded proteins from *C. jejuni* subsp. *jejuni* NCTC 11168 were used as query genes and proteins for identification enzymes involved in oxidative and nitrosative stress in *C. concisus* are listed in Additional file 9: Table S9. All additional references cited in additional files can be found in Additional file 12.

### Quantitative analysis of *C. concisus* growth on media supplemented with sodium fumarate under anaerobic conditions with and without hydrogen

We previously showed that sodium fumarate significantly increased the growth of *C. concisus* under anaerobic conditions in the presence of H_2_, supporting that fumarate is an electron acceptor in *C. concisus* [61]. To examine whether fumarate is an electron donor in *C. concisus*, in this study, we compared the growth of *C. concisus* under anaerobic conditions with and without H_2_ on media supplemented with sodium fumarate.

Three strains of *C. concis*us were randomly selected to examine their growth when sodium fumarate (Sigma-Aldrich, Missouri, USA) is supplemented in media and incubated in anaerobic conditions with and without hydrogen. These strains were P2CDO4, H17O-S1 and P26UCO-S; each are orally isolated *C. concisus* strains from a patient with Crohn’s disease, a healthy control and a patient with ulcerative colitis, respectively. Each strain was first cultured on HBA (Oxoid, Hampshire, UK) with 6% defibrinated horse blood and incubated in anaerobic conditions with 5% hydrogen as previously described [61]. Cultures were prepared to an optical density of 0.1 at a wavelength of 595 nm and 5 µL was inoculated onto two sets of HBA, HBA with 0.05% sodium fumarate (HBA^fum0.05^), HBA with 0.2% sodium fumarate (HBA^fum0.2^) and HBA with 0.4% sodium fumarate (HBA^fum0.4^). Each set of media was incubated in anaerobic conditions either with or without 5% hydrogen available. Plates were incubated for 48 h and CFU were subsequently quantified as previously described [61]. The CFU of the initial cell suspension used for inoculation was also quantified, allowing determination of *C. concisus* growth under cultivation conditions used [61]. Experiments were repeated three times.

### Examination of the effects of DMSO on the growth of *C. concisus*

The above three *C. concisus* strains were also used to examine whether DMSO affects the growth of *C. concisus*. *C. concisus* strains were cultured as described above. Cultures were prepared to optical density of 0.025 at a wavelength of 595 nm and 5 µL was inoculated onto two sets of HBA plates or HBA plates supplemented with 0.2% DMSO (ThermoFisher Scientific, Massachusetts, USA) for 48 h under anaerobic conditions containing 5% H_2_. CFUs were determined as described previously [61].

### Statistical analysis

The CFUs of *C. concisus* strains cultured on HBA plates supplemented with sodium fumarate and DMSO under different atmospheric conditions were compared using 2-tailed *t*-tests. A *P* value of less than 0.05 was considered significant.

## Supplementary information

**Additional file 1: Table S1.** NCBI locus tags for genes involved in central carbon metabolism.

**Additional file 2: Table S2.** NCBI locus tags for genes involved in use of electron donors of *C. concisus*

**Additional file 3: Table S3.** Query genes and proteins from *E. coli* strain K-12 MG1655 for identification of genes and proteins in *C. concisus* central carbon metabolism pathways.

**Additional file 4: Table S4.** Query genes and proteins from *C. jejuni* subsp. *jejuni* NCTC 11168 for identification of genes and proteins of *C. concisus* central carbon metabolism pathways.

**Additional file 5: Table S5.** NCBI locus tags for genes involved in use of electron acceptors.

**Additional file 6: Table S6.** NCBI locus tags for genes involved in amino acid use.

**Additional file 7: Table S7.** Query genes and proteins from *C. jejuni* subsp. *jejuni* NCTC 11168, *E. coli* strain K-12 MG1655, *and C. jejuni* subsp. *jejuni* strain 81116 used to identify genes and proteins for amino acid use in *C. concisus*.

**Additional file 8: Table S8.** NCBI locus tags for genes involved in oxidative stress.

**Additional file 9: Table S9.** Query genes and proteins from *C. jejuni* subsp. *jejuni* NCTC 11168 that were used to identify genes and proteins for oxidative and nitrosative stress defence in *C. concisus.*

**Additional file 10: Table S10.***C. concisus* strains used in BLASTn analysis.

**Additional file 11: Table S11.** Genes encoding electron donors and acceptors investigated in *C. concisus* as referenced from *C. jejuni* subsp. *jejuni* NCTC 11168 and *C. jejuni* subsp. *jejuni* strain 81116.

**Additional file 12.** Additional references cited in additional files 7, 9 and 11.

## Data Availability

All data generated or analysed during this study are included in this published article (and its additional information files).
