# Supplementary material for: Analyses of energy metabolism and stress defence provide insights into Campylobacter concisus growth and pathogenicity
Source: Gut Pathog. 2020 Mar 5;12:13. doi: 10.1186/s13099-020-00349-6 (PMC7059363; doi:10.1186/s13099-020-00349-6)
Supplement: Supplementary file 1 — Additional file 1: Table S1. NCBI locus tags for genes involved in central carbon metabolism. [file 13099_2020_349_MOESM1_ESM.pdf]

Analyses of energy metabolism and stress defence provide insights into *Campylobacter concisus* growth and pathogenicity

Table S1: NCBI locus tags for genes involved in central carbon metabolism

(Prefixes for locus tags are as follows: : *C. concisus* strain 13826: CCC13826_; *C. concisus* strain ATCC 33237: CCON33237_; *C. concisus* strain P2CDO4: CCS77_.)

Table S1a: NCBI locus tags for genes involved in the Embden Meyherhof Pathway of *C. concisus*

**EMP (compared with *E. coli* str. K-12 substr. MG1655)**															
	glk	***pgi***	***pfkA***	***pfkB***	***fbaA***	***fbaB***	***tpiA***	***gapA***	***pgk***	***gpmA***	***gpmM***	***ytjC***	***eno***	***pykA***	***pykF***
**13826**	**-**	**-**	**-**	**-**	**-**	**-**	**0514**	**0516**	**0515**	**-**	**0563**	**-**	**2093**	**0964**	**0964**
**ATCC 33237**	**-**	**-**	**-**	**-**	**-**	**-**	**1485**	**1487**	**1486**	**-**	**1496**	**-**	**1664**	**1542**	**1542**
**P2CDO4**	**-**	**-**	**-**	**-**	**-**	**-**	**1525**	**1527**	**1526**	**-**	**1555**	**-**	**1696**	**0396**	**0396**
**EMP *(*compared with *C. jejuni* subsp. *jejuni* NCTC 11168*)***															
	glk	***pgi***	***pfk***	***fba***	***tpiA***	***gapA***	***pgk***	***gpm***	***eno***	***pyk***					
**13826**	**n/a**	**0430**	**n/a**	**2070**	**0514**	**0516**	**0515**	**0563**	**2093**	**0964**					
**ATCC 33237**	**n/a**	**1355**	**n/a**	**1271**	**1485**	**1487**	**1486**	**1496**	**1664**	**1542**					
**P2CDO4**	**n/a**	**0594**	**n/a**	**1259**	**1525**	**1527**	**1526**	**1555**	**1696**	**0396**					

Table S1b: NCBI locus tags for genes involved in the Pentose Phosphate Pathway of *C. concisus*

PPP (compared with *E. coli* str. *K-12* substr. MG1655)											
	glk	*zwf*	*pgl*	*gnd*	*rpiA*	*rpiB*	*rpe*	*tktA*	*tktB*	*talA*	*talB*
13826	-	-	-	-	-	2228	1342	1735	1735	-	-
ATCC 33237	-	-	-	-	-	0591	0736	1715	1715	-	-
P2CDO4	-	-	-	-	-	1396	0916	1794	1794	-	-
PPP (compared with *C. jejuni* subsp. *jejuni* NCTC 11168)											
	glk	*zwf*	*pgl*	*gnd*	*rpiB*	*rpe*	*tkt*	*tal*			
13826	n/a	n/a	n/a	n/a	2228	1342	1735	1583			
ATCC 33237	n/a	n/a	n/a	n/a	0591	0736	1715	1442			
P2CDO4	n/a	n/a	n/a	n/a	1396	0916	1794	1422			

Table S1c: NCBI locus tags for genes involved in the Entner Doudouroff Pathway of *C. concisus*

ED Pathway (compared with *E. coli* str. K-12 substr. MG1655)							
	ptsG	*pgi*	*glk*	*pgl*	*zwf*	*edd*	*eda*
13826	-	-	-	-	-	-	-
ATCC 33237	-	-	-	-	-	-	-
P2CDO4	-	-	-	-	-	-	-
ED Pathway (compared with *C. jejuni* subsp. *doylei* 269.97)							
	glcP	*pgi*	*glk*	*pgl*	*zwf*	*edd*	*eda*
13826	-	0430	-	-	-	-	-
ATCC 33237	-	1355	-	-	-	-	-
P2CD04	-	0594	-	-	-	-	-

Table S1d: NCBI locus tags for genes involved in the tricarboxylic cycle of *C. concisus*

TCA cycle (compared with *E. coli* str. K12 substr. MG1655)																				
	gltA	*acnA*	*acnB*	*icd*	*sucA*	*sucB*	*sucC*	*sucD*	*sdhA*	*sdhB*	*sdhC*	*sdhD*	*mdh*	*mqo*	*fumA*	*fumB*	*fumC*	*lpdA*	*aceA*	*aceB*
13826	2287	-	1408	1088	-	-	-	-	0425	0424	-	-	2254	-	-	-	-	-	-	-
ATCC 33237	1642	-	0630	0995	-	-	-	-	1361	1362	-	-	0994	-	-	-	-	-	-	-
P2CDO4	1726	-	1110	1019	-	-	-	-	0589	0588	-	-	1018	-	-	-	-	-	-	-
TCA cycle (compared with *C. jejuni* subsp. *jejuni* NCTC 11168)																				
	gltA	*acnB*	*icd*	*oorA*	*oorB*	*oorC*	*oorD*	*sucC*	*sucD*	*frdA*	*frdB*	*frdC*	*mrfA*	*mrfB*	*mrfE*	*mdh*	*mqo*	*fumC*		
13826	2287	1408	1088	1294	1293	1292	1295	-	-	0425	0424	0426	1283	1282	1281	2254	0434	-		
ATCC 33237	1642	0630	0995	0992	0991	0990	0993	-	-	1361	1362	1360	0984	0983	0982	0994	1481	-		
P2CDO4	1726	1110	1019	1016	1015	1014	1017	-	-	0589	0588	0590	1012	1011	1010	1018	1519	-		

Table S1e: NCBI locus tags for genes involved in the pyruvate dehydrogenase complex of *C. concisus*

Pyruvate Dehydrogenase Complex (compared with *E. coli* str. K-12 substr. MG1655)				
	aceE	*aceF*	*lpdA*	*pfo/ydbK*
13826	-	-	-	1933
ATCC 33237	-	-	-	1610
P2CDO4	-	-	-	0237
Pyruvate-Flavodoxin Oxidoreductase (compared with *C. jejuni* subsp. *jejuni* NCTC 11168)				
	por			
13826	1933			
ATCC 33237	1610			
P2CDO4	0237			

Table S1f: NCBI locus tags for genes involved in acetate metabolism of *C. concisus*

**Acetate metabolism (compared with *E. coli* str. K-12 substr. MG1655)**			
	acka	***pta***	***acs***
**13826**	**0104**	**0103**	**-**
**ATCC 33237**	**1014**	**0104**	**-**
**P2CDO4**	**1039**	**1038**	**-**
**Acetate metabolism (compared with *C. jejuni* subsp . *jejuni* NCTC 11168)**			
	cj0689	***cj1688***	***cj1537c***
**13826**	**0104**	**0103**	**-**
**ATCC 33237**	**1014**	**0104**	**-**
**P2CDO4**	**1039**	**1038**	**-**

Table S1g: NCBI locus tags for genes involved in gluconeogenesis of *C. concisus*

**Gluconeogenesis (compared with *C. jejuni* subsp . *jejuni* NCTC 11168)**				
	pckA	***fbp***	***pycA***	***pycB***
**13826**	**1509**	**1544**	**1812**	**1508**
**ATCC 33237**	**0495**	**0574**	**0148**	**0494**
**P2CDO4**	**0496**	**1457**	**0067**	**0495**
