# Supplementary material for: Analyses of energy metabolism and stress defence provide insights into Campylobacter concisus growth and pathogenicity
Source: Gut Pathog. 2020 Mar 5;12:13. doi: 10.1186/s13099-020-00349-6 (PMC7059363; doi:10.1186/s13099-020-00349-6)
Supplement: Supplementary file 2 — Additional file 2: Table S2. NCBI locus tags for genes involved in use of electron donors of C. concisus [file 13099_2020_349_MOESM2_ESM.pdf]

Analyses of energy metabolism and stress defence provide insights into *Campylobacter concisus* growth and pathogenicity

Table S2: NCBI locus tags for genes involved in use of electron donors of *C. concisus*

Table S2a: NCBI locus tags for genes involved in use of electron donors of *C. concisus* (2-oxoglutarate, Flavodoxin)

(Prefixes for locus tags are as follows: *C. concisus* strain 13826: CCC13826_; *C. concisus* strain ATCC 33237: CCON33237_; *C. concisus strain* P2CDO4: CCS77_.)

	2-oxo-glutarate				Flavodoxin														
**Gene (with *C. jejuni* subsp. *jejuni* NCTC 1168 as reference)**	***cj0536 oorA***	***cj0537 oorB***	***cj0538 oorC***	***cj0535 oorD***	***cj1579c nuoA***	***cj1578c nuoB***	***cj1577c nuoC***	***cj1576c nuoD***	***cj1575c***	***cj1574c***	***cj1573c nuoG***	***cj1572c nuoH***	***cj1571c nuoI***	***cj1570c nuoJ***	***cj1569c nuoK***	***cj1568c nuoL***	***cj1567c nuoM***	***cj1566c nuoN***	***cj1382c fldA***
***C. concisus* 13826**	**1294**	**1293**	**1292**	**1295**	**1656**	**1657**	**1658**	**1659**	**1660**	**1661**	**1662**	**1663**	**1664**	**1665**	**1666**	**1667**	**1668**	**1669**	**1656**
***C. concisus* ATCC 33237**	**0992**	**0991**	**0990**	**0993**	**0216**	**0215**	**0214**	**0213**	**0212**	**0211**	**0210**	**0209**	**0208**	**0207**	**0206**	**0205**	**0204**	**0203**	**1606**
***C. concisus* P2CDO4**	**1016**	**1015**	**1014**	**1017**	**0213**	**0214**	**0215**	**0216**	**0217**	**0218**	**0219**	**0220**	**0221**	**0222**	**0223**	**0224**	**0225**	**0226**	**0241**

Table S2b: NCBI locus tags for genes involved in use of electron donors of *C. concisus* (Formate, Fumarate, Gluconate, Hydrogen)

	Formate				Fumarate	Gluconate				Hydrogen										
**Gene (with *C. jejuni* subsp. *jejuni* NCTC 1168 as reference)**	***cj1511c fdhA***	***cj1510c fdhB***	***cj1509c fdhC***	***cj1508c fdhD***	***cj1364c fumC***	***cj0414***	***cj0415***	****edd JJD26997_1271***	****eda JJD26997_1272***	***cj1267c hydA***	***cj1266c hydB***	***cj1265c hydC***	***cj1264c hydD***	***cj0627 hypA***	***cj0623 hypB***	***cj0624 hypC***	***cj0625 hypD***	***cj0626 hypE***	***cj0622 hypF***	***cj1584c nikZ***
***C. concisus* 13826**	**0207**	**0762**	**2138**	**0211**	**-**	**-**	**-**	**-**	**-**	**0101**	**0100**	**0099**	**0098**	**1092**	**1096**	**1095**	**1094**	**1093**	**0356**	**2116**
***C. concisus* ATCC 33237**	**0355**	**0356**	**0328**	**0548**	**-**	**-**	**-**	**-**	**-**	**1011**	**1010**	**1009**	**1008**	**0998**	**1002**	**1001**	**1000**	**0999**	**1006**	**0279**
***C. concisus* P2CDO4**	**0310**	**0311**	**1653**	**1566**	**-**	**-**	**-**	**-**	**-**	**1036**	**1035**	**1034**	**1033**	**1022**	**1026**	**1025**	**1024**	**1023**	**1030**	**1674**

Table S2c: NCBI locus tags for genes involved in use of electron donors of *C. concisus* (Lactate, Malate, Pyruvate, Succinate and Sulfite)

	Lactate				Malate		Pyruvate	Succinate			Sulphite	
**Gene (with *C. jejuni* subsp. *jejuni* NCTC 11168 as reference)**	***cj0073 lutC***	***cj0074 lutB***	***cj0075 lutA***	***cj1585c***	***cj0393c mqo***	***cj0532 mdh***	***cj1476c por***	***cj0409 frdA***	***cj0410 frdB***	***cj0408 frdC***	***cj0004c sorA***	***cj0005c sorB***
***C. concisus* 13826**	**-**	**-**	**-**	**-**	**0434**	**2254**	**1933**	**0425**	**0424**	**0426**	**-**	**-**
***C. concisus* ATCC 33237**	**-**	**-**	**-**	**-**	**1481**	**0994**	**1610**	**1361**	**1362**	**1360**	**-**	**-**
***C. concisus* P2CD04**	**-**	**-**	**-**	**-**	**1519**	**1018**	**0237**	**0589**	**0588**	**0590**	**-**	**-**
