# Supplementary material for: Analyses of energy metabolism and stress defence provide insights into Campylobacter concisus growth and pathogenicity
Source: Gut Pathog. 2020 Mar 5;12:13. doi: 10.1186/s13099-020-00349-6 (PMC7059363; doi:10.1186/s13099-020-00349-6)
Supplement: Supplementary file 3 — Additional file 3: Table S3. Query genes and proteins from E. coli strain K-12 MG1655 for identification of genes and proteins in C. concisus central carbon metabolism pathways. [file 13099_2020_349_MOESM3_ESM.pdf]

Analyses of energy metabolism and stress defence provide insights into *Campylobacter concisus* growth and pathogenicity

Table S3: Query genes and proteins from *E. coli* strain K-12 MG1655 for identification of genes and proteins in *C. concisus* central carbon metabolism pathways

Gene name	Locus tag	Relevant pathway	Protein Function
glk	*b2388*	EMP, PP and ED pathways	glucokinase phosphorylates glucose to glucose-6-phosphate
pgi	*b4025*	EMP and ED pathways	glucose-6-phosphate isomerase converts glucose-6-phosphate to fructose-6-phosphate
pfkA	*b3916*	EMP pathway	phosphofructokinase phosphorylates fructose-6-phosphate to fructose-1,6-bisphosphate
pfkB	*b1723*	EMP pathway	phosphofructokinase phosphorylates fructose-6-phosphate to fructose-1,6-bisphosphate
fbaA	*b2925*	EMP pathway	fructose-bisphosphate aldolase condenses dihydroxyacetone phosphate with glyceraldehyde-3-phosphate to form fructose bisphosphate
fbaB	*b2097*	EMP pathway	fructose-bisphosphate aldolase condenses dihydroxyacetone phosphate with glyceraldehyde-3-phosphate to form fructose bisphosphate
tpiA	*b3919*	EMP pathway	triosephosphate isomerase converts dihydroxyacetone phosphate to glyceraldehyde-3-phosphate
gapA	*b1179*	EMP pathway	glyceraldehyde-3-phosphate dehydrogenase A phosphorylates glyceraldehyde-3-phosphate to 1,3-bisphosphoglycerate
pgk	*b2926*	EMP pathway	phosphoglucokinase reversibly phosphorylates 3-phosphoglycerate to 1,3-bisphosphoglycerate
gpmA	*b0755*	EMP pathway	phosphoglycerate mutase interconverts 3-phosphoglycerate and 2-phosphoglycerate
gpmM	*b3612*	EMP pathway	phosphoglycerate mutase interconverts 3-phosphoglycerate and 2-phosphoglycerate
ytjC/ gpmB	*b4395*	EMP pathway	phosphoglycerate mutase interconverts 3-phosphoglycerate and 2-phosphoglycerate
eno	*b2779*	EMP pathway	enolase reversibly converts 2-phosphoglycerate to phosphoenolpyruvate
pykA	*b1854*	EMP pathway	pyruvate kinase phosphorylates phosphoenolpyruvate to pyruvate
pykF	*b1676*	EMP pathway	pyruvate kinase phosphorylates phosphoenolpyruvate to pyruvate
zwf	*b1852*	PP and ED pathways	glucose-6-phosphate 1-dehydrogenase oxidizes glucose-6-phosphate to 6-phosphoglucono-lactone
pgl	*b0767*	PP and ED pathways	6-phosphogluconolactonase hydrolyzes 6-phosphogluconolactone to 6-phosphogluconate
gnd	*b2029*	PP pathway	6-phosphogluconate dehydrogenase oxidatively decarboxylates 6-phosphogluconate to ribulose 5-phosphate and CO_2
rpiA	*b2914*	PP pathway	ribose-5-phosphate isomerase reversibly converts ribose-5-phosphate to ribulose 5-phosphate
rpiB	*b4090*	PP pathway	ribose-5-phosphate isomerase reversibly converts ribose-5-phosphate to ribulose 5-phosphate

rpe	*b3386*	PP pathway	ribulose-phosphate 3-epimerase reversibly epimerizes of ribulose-5-phosphate to xylulose 5-phosphate
tktA	*b2935*	PP pathway	transketolase converts sedoheptulose-7-phosphate and glyceraldehyde-3-phosphate to xylulose-5-phosphate and ribose-5-phosphate
tktB	*b2465*	PP pathway	transketolase converts sedoheptulose-7-phosphate and glyceraldehyde-3-phosphate to xylulose-5-phosphate and ribose-5-phosphate
talA	*b2464*	PP pathway	transladolase converts glyceraldehyde-3-phosphate and sedoheptulose 7-phosphate to fructose-6-phosphate and erythrose 4-phosphate
talB	*b0008*	PP pathway	transladolase converts glyceraldehyde-3-phosphate and sedoheptulose 7-phosphate to fructose-6-phosphate and erythrose 4-phosphate
ptsG	*b1101*	ED pathway	glucose permease imports glucose into the cell
edd	*b1851*	ED pathway	phosphogluconate dehydratase converts 6-phosphogluconate to 2-keto-3-deoxy-6-phosphogluconate
eda	*b1850*	ED pathway	2-dehydro-3-deoxy-phosphogluconate aldolase converts 2-dehydro-3-deoxy-D-gluconate 6-phosphate to glyceraldehyde-3-phosphate and pyruvate
gltA	*b0720*	TCA cycle	citrate synthase condenses acetyl-coA and oxaloacetate to citrate
acnA	*b1276*	TCA cycle	aconitase isomerizes citrate to isocitrate
acnB	*b0118*	TCA cycle	aconitase isomerizes citrate to isocitrate
ybhJ	*b0771*	TCA cycle	aconitase isomerizes citrate to isocitrate
icd	*b1136*	TCA cycle	isocitrate dehydrogenase catalyzes oxidative decarboxylation of isocitrate, to 2-oxoglutarate and CO_2
sucA	*b0726*	TCA cycle	2-oxoglutarate dehydrogenase converts 2-oxoglutarate to succinyl-CoA and CO_2
sucB	*b0727*	TCA cycle	2-oxoglutarate dehydrogenase converts 2-oxoglutarate to succinyl-CoA and CO_2
sucC	*b0728*	TCA cycle	succinyl-CoA synthetase catalyzes the reversible conversion of succinyl-CoA to succinate
sucD	*b0729*	TCA cycle	succinyl-CoA synthetase catalyzes the reversible conversion of succinyl-CoA to succinate
sdhA	*b0723*	TCA cycle	succinate dehydrogenase converts succinate to fumarate with the reduction of ubiquinone to ubiquinol
sdhB	*b0724*	TCA cycle	succinate dehydrogenase converts succinate to fumarate with the reduction of ubiquinone to ubiquinol
sdhC	*b0721*	TCA cycle	succinate dehydrogenase converts succinate to fumarate with the reduction of ubiquinone to ubiquinol
sdhD	*b0722*	TCA cycle	succinate dehydrogenase converts succinate to fumarate with the reduction of ubiquinone to ubiquinol
mdh	*b3236*	TCA cycle	malate dehydrogenase reversibly catalyzes the oxidation of malate to oxaloacetate
mqo	*b2210*	TCA cycle	malate quinone oxidoreductase reversibly catalyzes the oxidation of malate to oxaloacetate
fumA	*b1612*	TCA cycle	fumarate dehydratase reversibly converts fumarate to

			malate
fumB	*b4122*	TCA cycle	fumarate dehydratase reversibly converts fumarate to malate
fumC	*b1611*	TCA cycle	fumarate dehydratase reversibly converts fumarate to malate
lpdA	*b0116*	TCA cycle	dihydrolipoyl dehydrogenase converts pyruvate to acetyl-CoA and CO_2
aceA	*b4015*	Glyoxylate cycle	isocitrate lyase catalyzes the cleavage of isocitrate to succinate and glyoxylate
aceB	*b4014*	Glyoxylate cycle	malate synthase converts acetyl-CoA and glyoxylate to malate and CoA
aceE	*b0114*	TCA cycle	pyruvate dehydrogenase converts pyruvate to acetyl-CoA and CO_2
aceF	*b0115*	TCA cycle	pyruvate dehydrogenase converts pyruvate to acetyl-CoA and CO_2
ydbk/pfo	*b1378*	TCA cycle	pyruvate-flavodoxin oxidoreductase converts pyruvate to acetyl coA
ackA	*b2296*	Acetate metabolism	acetate kinase phosphorylates acetate to acetyl phosphate
pta	*b2297*	Acetate metabolism	phosphate acetyltransferase converts acetyl-CoA and phosphate to CoA and acetyl phosphate
acs	*b4069*	Acetate metabolism	acetyl CoA synthetase reversibly converts acetyl CoA to acetate
