# Supplementary material for: Analyses of energy metabolism and stress defence provide insights into Campylobacter concisus growth and pathogenicity
Source: Gut Pathog. 2020 Mar 5;12:13. doi: 10.1186/s13099-020-00349-6 (PMC7059363; doi:10.1186/s13099-020-00349-6)
Supplement: Supplementary file 4 — Additional file 4: Table S4. Query genes and proteins from C. jejuni subsp. jejuni NCTC 11168 for identification of genes and proteins of C. concisus central carbon metabolism pathways. [file 13099_2020_349_MOESM4_ESM.pdf]

Analyses of energy metabolism and stress defence provide insights into *Campylobacter concisus* growth and pathogenicity

Table S4: Query genes and proteins from *C. jejuni* subsp. *jejuni* NCTC 11168 for identification of genes and proteins of *C. concisus* central carbon metabolism pathways

Gene name	Locus tag	Relevant pathway	Protein Function
*glk**	*JJD26997_ 1268*	EMP, PP and ED pathways	glucokinase phosphorylates glucose to glucose-6-phosphate
*pgi**	*cj1535c/ JJD26997_ 1267*	EMP and ED pathways	glucose-6-phosphate isomerase converts glucose-6-phosphate to fructose-6-phosphate
fba	*cj0597*	EMP pathway	fructose-bisphosphate aldolase condenses dihydroxyacetone phosphate with glyceraldehyde-3-phosphate to form fructose bisphosphate
tpiA	*cj401c*	EMP pathway, gluconeogenesis	triosephosphate isomerase converts dihydroxyacetone phosphate to glyceraldehyde-3-phosphate
gapA	*cj1403c*	EMP pathway	glyceraldehyde-3-phosphate dehydrogenase A phosphorylates glyceraldehyde-3-phosphate to 1,3-bisphosphoglycerate
pgk	*cj1402c*	EMP pathway, gluconeogenesis	phosphoglucokinase reversibly phosphorylates 1,3-bisphosphoglycerate to 3-phosphoglycerate
pgm	*cj0434*	EMP pathway	Phosphoglycerate mutase interconverts 3-phosphoglycerate and 2-phosphoglycerate
eno	*cj1672c*	EMP pathway	Enolase reversibly converts 2-phosphoglycerate to phosphoenolpyruvate
pyk	*cj0392c*	EMP pathway	pyruvate kinase phosphorylates phosphoenolpyruvate to pyruvate
*zwf**	*JJD26997_ 1270*	PP and ED pathways	glucose-6-phosphate 1-dehydrogenase oxidizes glucose-6-phosphate to 6-phosphoglucono-lactone
*pgl**	*JJD26997_ 1269*	PP and ED pathways	6-phosphogluconolactonase hydrolyzes 6-phosphogluconolactone to 6-phosphogluconate
rpiB	*cj0925*	PP pathway	ribose-5-phosphate isomerase reversibly converts ribose-5-phosphate to ribulose 5-phosphate
rep	*cj0451*	PP pathway	ribulose-phosphate 3-epimerase reversibly epimerizes of ribulose 5-phosphate to xylulose 5-phosphate
tkt	*cj1645*	PP pathway	transketolase converts sedoheptulose-7-phosphate and glyceraldehyde-3-phosphate to xylulose-5-phosphate and ribose-5-phosphate
tal	*cj0281c*	PP pathway	transladolase converts glyceraldehyde-3-phosphate and sedoheptulose 7-phosphate to fructose-6-phosphate and erythrose 4-phosphate
*glcP**	*JJD26997_ 1266*	ED pathway	glucose permease imports glucose into the cell
*edd **	*JJD26997_ 1271*	ED pathway	phosphogluconate dehydratase converts 6-phosphogluconate to 2-keto-3-deoxy-6-phosphogluconate
*eda **	*JJD26997_*	ED pathway	2-dehydro-3-deoxy-phosphogluconate aldolase

	1272		converts 2-dehydro-3-deoxy-D-gluconate 6-phosphate to glyceraldehyde-3-phosphate and pyruvate
gltA	*cj1682*	TCA cycle	citrate synthase condenses acetyl-coA and oxaloacetate to citrate
acnB	*cj0835c*	TCA cycle	aconitase isomerizes citrate to isocitrate
icd	*cj0531*	TCA cycle	isocitrate dehydrogenase catalyzes oxidative decarboxylation of isocitrate, to 2-oxoglutarate and CO_2
oorA	*cj0536*	TCA cycle	2-oxoglutarate oxidoreductase converts 2-oxo-glutarate to succinyl-coA and CO_2
oorB	*cj0537*	TCA cycle	2-oxoglutarate oxidoreductase converts 2-oxo-glutarate to succinyl-coA and CO_2
oorC	*cj0538*	TCA cycle	2-oxoglutarate oxidoreductase converts 2-oxo-glutarate to succinyl-coA and CO_2
oorD	*cj0539*	TCA cycle	2-oxoglutarate oxidoreductase converts 2-oxo-glutarate to succinyl-coA and CO_2
sucC	*cj0533*	TCA cycle	succinyl-CoA synthetase catalyzes the reversible conversion of succinyl-CoA to succinate
sucD	*cj0534*	TCA cycle	succinyl-CoA synthetase catalyzes the reversible conversion of succinyl-CoA to succinate
mrfA	*cj0437*	TCA cycle	methylmenaquinol fumarate reductase converts fumarate to succinate with the oxidation of menaquinol to menaquinone
mrfB	*cj0438*	TCA cycle	methylmenaquinol fumarate reductase converts fumarate to succinate with the oxidation of menaquinol to menaquinone
mrfE	*cj0439*	TCA cycle	methylmenaquinol fumarate reductase converts fumarate to succinate with the oxidation of menaquinol to menaquinone
frdA	*cj0409*	TCA cycle	bidirectional fumarate reductase reversibly converts succinate to fumarate with the reduction of menaquinone to menaquinol
frdB	*cj0410*	TCA cycle	bidirectional fumarate reductase reversibly converts succinate to fumarate with the reduction of menaquinone to menaquinol
frdC	*cj0408*	TCA cycle	bidirectional fumarate reductase reversibly converts succinate to fumarate with the reduction of menaquinone to menaquinol
mdh	*cj0532*	TCA cycle	malate dehydrogenase reversibly catalyzes the oxidation of malate to oxaloacetate
mqo	*cj0393c*	TCA cycle	malate quinone oxidoreductase reversibly catalyzes the oxidation of malate to oxaloacetate
fumC	*cj1364c*	TCA cycle	fumarate hydratase reversibly converts fumarate to malate
ackA	*cj0689*	Acetate metabolism	acetate kinase phosphorylates acetate to acetyl phosphate
pta	*cj0688*	Acetate metabolism	phosphate acetyltransferase converts acetyl-CoA and phosphate to CoA and acetyl phosphate
acs	*cj1537c*	Acetate metabolism	acetyl CoA synthetase reversibly converts acetyl CoA to acetate
pycA	*cj1037c*	Gluconeogenesis	pyruvate carboxylase subunit A involved in conversion of pyruvate to oxaloacetate
pycB	*cj1038c*	Gluconeogenesis	pyruvate carboxylase subunit B involved in

			conversion of pyruvate to oxaloacetate
pckA	*cj0932c*	Gluconeogenesis	phosphoenolpyruvate carboxykinase converts oxaloacetate to phosphoenolpyruvate
fbp	*cj00840c*	Gluconeogenesis	fructose-1,6-bisphosphatase converts fructose-1,6-bisphosphate to D-fructose 6-phosphate
