# Supplementary material for: Analyses of energy metabolism and stress defence provide insights into Campylobacter concisus growth and pathogenicity
Source: Gut Pathog. 2020 Mar 5;12:13. doi: 10.1186/s13099-020-00349-6 (PMC7059363; doi:10.1186/s13099-020-00349-6)
Supplement: Supplementary file 5 — Additional file 5: Table S5. NCBI locus tags for genes involved in use of electron acceptors. [file 13099_2020_349_MOESM5_ESM.pdf]

Analyses of energy metabolism and stress defence provide insights into *Campylobacter concisus* growth and pathogenicity

Table S5: NCBI locus tags for genes involved in use of electron acceptors

(Prefixes for locus tags are as follows: *C. concisus* strain 13826: CCC13826_; *C. concisus* strain ATCC 33237: CCON33237_; *C. concisus* strain P2CDO4: CCS77_.)

Table S5: NCBI locus tags for genes involved in use of electron acceptors of *C. concisus* (Fumarate, Nitrate, Nitrite)

	**Fumarate**			**Nitrate**						**Nitrite**	
**Gene (with *C. jejuni* subsp. *jejuni* 11168 as reference)**	***cj0437 mrfA***	***cj0438 mrfB***	***cj0439 mrfE***	***cj0780 napA***	***cj0781 napG***	***cj0782 napH***	***cj0783 napB***	***cj0784 napL***	***cj0785 napD***	***cj1357c nrfA***	***cj1358c nrfH***
***C. concisus* 13826**	**1283**	**1282**	**1281**	**0868**	**0867**	**0866**	**0865**	**0863**	**0862**	**-**	**-**
***C. concisus* ATCC 33237**	**0984**	**0983**	**0982**	**0641**	**0642**	**0643**	**0644**	**0646**	**0647**	**-**	**-**
***C. concisus* P2CD04**	**1012**	**1011**	**1010**	**0645**	**0646**	**0647**	**0648**	**0650**	**0651**	**-**	**-**

Table S5b: NCBI locus tags for genes involved in use of electron acceptors of *C. concisus* (Oxygen, SN-oxide, Tetrathionate)

	**Oxygen**									**SN-Oxide**		**Tetrathionate**	
**Gene (with *C. jejuni* subsp. *jejuni* 11168 as reference)**	***cj1490c ccoN***	***cj1489c ccoO***	***cj1488c ccoQ***	***cj1487c ccoP***	***cj1186c petA***	***cj1185c petB***	***cj1184c petC***	***cj0081 cioA***	***cj0082 cioB***	***cj0264c torA***	***cj0265c torC***	***C8J_0815 tsdA***	***C8J_0040***
***C. concisus* 13826**	**0723**	**0724**	**0725**	**0299**	**1921**	**1920**	**1919**	**0906**	**0905**	**1119**	**1398**	**-**	**-**
***C. concisus* ATCC 33237**	**0296**	**0297**	**0298**	**0726**	**1599**	**1598**	**1597**	**1795**	**1796**	**0015**	**0067**	**-**	**-**
***C. concisus* P2CD04**	**0272**	**0273**	**0274**	**0275**	**0249**	**0250**	**0251**	**1880**	**1881**	**1098**	**1099**	**-**	**-**
