# Supplementary material for: Analyses of energy metabolism and stress defence provide insights into Campylobacter concisus growth and pathogenicity
Source: Gut Pathog. 2020 Mar 5;12:13. doi: 10.1186/s13099-020-00349-6 (PMC7059363; doi:10.1186/s13099-020-00349-6)
Supplement: Supplementary file 6 — Additional file 6: Table S6. NCBI locus tags for genes involved in amino acid use. [file 13099_2020_349_MOESM6_ESM.pdf]

Analyses of energy metabolism and stress defence provide insights into *Campylobacter concisus* growth and pathogenicity

Table S6: NCBI locus tags for genes involved in amino acid use

(Prefixes for locus tags are as follows: : *C. concisus* strain 13826: CCC13826_; *C. concisus* strain ATCC 33237: CCON33237_; *C. concisus* strain P2CDO4: CCS77_.)

Table S6a: NCBI locus tags for genes involved in use of amino acids of *C. concisus*

**Amino acid use (compared with *C. jejuni* subsp. *jejuni* NCTC 11168)**													
	**Aspartate**				**Asparagine**	**Glutamate**							
	dcuA	***dcuB***	***dctA***	***aspA***	***ansB***	***cj0919c***	***cj0920c***	***cj0921c***	***cj0922c***	***gdhA***	***paqP***	***paqQ***	***aspB***
**13826**	**0390**	**1000**	**1512**	**0391**	**0029**	**0415**	**1927**	**0664**	**0414**	**1815**	**0661**	**0663**	**1274**
**ATCC 33237**	**1351**	**0438**	**0496**	**1352**	**1259**	**1381**	**1604**	**1189**	**1380**	**0145**	**1177**	**0319**	**0913**
**P2CDO4**	**0598**	**0430**	**0499**	**0597**	**1244**	**1435**	**0243**	**0762**	**1434**	**0064**	**0765**	**0763**	**1003**

Table S6b: NCBI locus tags for genes involved in use of amino acids of *C. concisus*

**Amino acid use (compared with *C. jejuni* subsp . *jejuni* NCTC 11168)**										
	**Serine**		**Proline**		**Isoleucine, Leucine, Valine**					
	sdaA	***sdaC***	***putA***	***putP***	***livJ***	***livK***	***livH***	***livM***	***livG***	***livF***
**13826**	**-**	**-**	**-**	**0416**	**-**	**-**	**-**	**-**	**-**	**-**
**ATCC 33237**	**-**	**-**	**-**	**1382**	**-**	**-**	**-**	**-**	**-**	**-**
**P2CDO4**	**-**	**-**	**-**	**1436**	**-**	**-**	**-**	**-**	**-**	**-**
