# Supplementary material for: Analyses of energy metabolism and stress defence provide insights into Campylobacter concisus growth and pathogenicity
Source: Gut Pathog. 2020 Mar 5;12:13. doi: 10.1186/s13099-020-00349-6 (PMC7059363; doi:10.1186/s13099-020-00349-6)
Supplement: Supplementary file 7 — Additional file 7: Table S7. Query genes and proteins from C. jejuni subsp. jejuni NCTC 11168, E. coli strain K-12 MG1655, and C. jejuni subsp. jejuni strain 81116 used to identify genes and proteins for amino acid use in C. concisus. [file 13099_2020_349_MOESM7_ESM.pdf]

Analyses of energy metabolism and stress defence provide insights into *Campylobacter concisus* growth and pathogenicity

Table S7: Query genes and proteins from *C. jejuni* subsp. *jejuni* NCTC 11168, *E. coli* strain K-12 MG1655, *and C. jejuni* subsp. *jejuni* strain 81116 used to identify genes and proteins for amino acid use in *C. concisus*

Gene name	*Locus tag*	Function	Reference
sdaA	*cj1624c*	serine dehydratase converts serine to pyruvate	1
sdaC	*cj1625c*	transports serine	1
putA	*cj1503c*	proline dehydrogenase converts proline to S-1-pyrroline carboxylate	2
putP	*cj1502c*	transports proline	2
dcuA	*cj0088*	uptake of fumarate and aspartate	3
dcuB	*cj0671*	uptake of fumarate and aspartate, succinate efflux	3
dctA	*cj1192*	uptake of fumarate, aspartate and succinate	3
aspA	*cj0087*	aspartate ammonia lyase converts aspartate to fumarate and ammonia	4
aspB	*cj0762c*	aspartate:glutamate transaminase converts glutamate and oxaloacetate to 2-oxoglutarate and aspartate	4
ansB	*cj0029*	asparaginase deaminates asparagine to aspartate	5
	cj0919c	ABC-type amino-acid transporter permease	6
	cj0920c	ABC-type amino-acid transporter permease	6
peb1a	*cj0921c*	aspartate/glutamate-binding protein of ABC transporter	6
pebC	*cj0922c*	ABC-type amino-acid transporter ATP-binding protein	6
paqP	*cj0467*	ABC-type glutamate transporter permease	7
paqQ	*cj0468*	ABC-type glutamate transpoter ATP-binding protein	7
gdhA	*b1761*	glutamate dehydrogenase converts glutamate to 2-oxoglutarate	8
livJ	*CJJ81176_1038*	periplasmic binding protein for branched chain amino acid transport	9
livK	*CJJ81176_1037*	periplasmic binding protein for branched chain amino acid transport	9
livH	*CJJ81176_1036*	inner membrane permease for branched chain amino acid transport	9
livM	*CJJ81176_1035*	inner membrane permease for branched chain amino acid transport	9
livG	*CJJ81176_1034*	cytoplasmic ATPase for branched chain amino acid transport	9
livF	*CJJ81176_1033*	cytoplasmic ATPase for branched chain amino acid transport	9
