# Supplementary material for: Analyses of energy metabolism and stress defence provide insights into Campylobacter concisus growth and pathogenicity
Source: Gut Pathog. 2020 Mar 5;12:13. doi: 10.1186/s13099-020-00349-6 (PMC7059363; doi:10.1186/s13099-020-00349-6)
Supplement: Supplementary file 8 — Additional file 8: Table S8. NCBI locus tags for genes involved in oxidative stress. [file 13099_2020_349_MOESM8_ESM.pdf]

Analyses of energy metabolism and stress defence provide insights into *Campylobacter concisus* growth and pathogenicity

Table S8: NCBI locus tags for genes involved in oxidative stress

(Prefixes for locus tags are as follows: *C. concisus* strain 13826: CCC13826_; *C. concisus* strain ATCC 33237: CCON33237_; *C. concisus* strain P2CDO4: CCS77_.)

Additional Table S8: NCBI locus tags for genes involved in use oxidative stress defense mechanisms of *C. concisus*

	ahpC	***bcp***	***tpx***	***docA/cj0358***	***dps***	***katA***	***mdaB***	***msrA/B***	***rrc***	***sodB***
**13826**	**0985**	**2112**	**0323**	**1108**	**1456**	**-**	**1626**	**1633**	**1910**	**0328**
**ATCC 33237**	**1523**	**0275**	**1761**	**0161**	**0695**	**-**	**0755**	**0761**	**1802**	**1766**
**P2CD04**	**0419**	**1678**	**1855**	**0114**	**0694**	**-**	**0798**	**0805**	**1901**	**1849**
