# Supplementary material for: Analyses of energy metabolism and stress defence provide insights into Campylobacter concisus growth and pathogenicity
Source: Gut Pathog. 2020 Mar 5;12:13. doi: 10.1186/s13099-020-00349-6 (PMC7059363; doi:10.1186/s13099-020-00349-6)
Supplement: Supplementary file 9 — Additional file 9: Table S9. Query genes and proteins from C. jejuni subsp. jejuni NCTC 11168 that were used to identify genes and proteins for oxidative and nitrosative stress defence in C. concisus. [file 13099_2020_349_MOESM9_ESM.pdf]

Analyses of energy metabolism and stress defence provide insights into *Campylobacter concisus* growth and pathogenicity

Table S9: Query genes and proteins from *C jejuni* subsp. *jejuni* NCTC 11168 that were used to identify genes and proteins for oxidative and nitrosative stress defence in *C. concisus*

Gene name	*Locus tag*	Function	Reference
docA	*cj0020c*	cytochrome C peroxidase with unknown specificity	10
	cj0358	cytochrome C peroxidase with unknown specificity	10
ahpC	*cj0334*	alkyl hydroperoxide reductase is involved in oxidative stress defence possibly from an endogenous organic peroxide	11
bcp	*cj0271*	bacterioferritin comigratory protein acts as a peroxide reductase able to act on a wide variety of compounds including hydrogen peroxide and organic peroxides	12
dps	*cj1534c*	DNA-binding protein involved in sequestration of Fe ions	13, 14, 15
katA	*cj1385*	catalase converts hydrogen peroxide into water and oxygen	13
mdaB	*cj1545c*	reduction of soluble quinones in *Helicobacter pylori*	13, 16
msrA	*cj0637c*	S-isomer specific methionine sulphoxide reductase reduces oxidized S-methionine	17
msrB	*cj1112c*	R-isomer specific methionine sulphoxide reductase reduces oxidized R-methionine	17
rrc	*cj0012c*	desulforubrerythrin is involved in hydrogen peroxide detoxification	13
sodB	*cj0169*	superoxide dismutase converts superoxide to hydrogen peroxide and oxygen	13
tpx	*cj0779*	thiol peroxidase reduces hydrogen peroxide	12
cgb	*cj1586*	single domain hemoglobin involved in nitric oxide detoxification	18
ctb	*cj0465c*	truncated hemoglobin involved in nitric oxide detoxification	19
nrfA	*cj1357c*	nitrite reductase reduces nitrite to ammonia, and is involved in nitric oxide detoxification	20
