# Supplementary material for: Analyses of energy metabolism and stress defence provide insights into Campylobacter concisus growth and pathogenicity
Source: Gut Pathog. 2020 Mar 5;12:13. doi: 10.1186/s13099-020-00349-6 (PMC7059363; doi:10.1186/s13099-020-00349-6)
Supplement: Supplementary file 10 — Additional file 10: Table S10.C. concisus strains used in BLASTn analysis. [file 13099_2020_349_MOESM10_ESM.pdf]

Analyses of energy metabolism and stress defence provide insights into *Campylobacter concisus* growth and pathogenicity

Table S10: *Campylobacter concisus* strains used in BLASTn analysis

	**Strain Name**	Genome Status
1	13826	Fully sequenced
2	2009-118452	Draft genome
3	2009-119100	Draft genome
4	2009-129008	Draft genome
5	2009-130586	Draft genome
6	2009-158448	Draft genome
7	2009-173039	Draft genome
8	2009-42653	Draft genome
9	2009-75710	Draft genome
10	2009-75775	Draft genome
11	2009-86120	Draft genome
12	2009-91522	Draft genome
13	2010-112100-F	Draft genome
14	2010-112100-O	Draft genome
15	2010-112708	Draft genome
16	2010-112758	Draft genome
17	2010-112825	Draft genome
18	2010-113332-F	Draft genome
19	2010-113332-O	Draft genome
20	2010-113862	Draft genome
21	2010-113862-O	Draft genome
22	2010-115605-F	Draft genome
23	2010-131105	Draft genome
24	2010-16206	Draft genome
25	2010-164712	Draft genome
26	2010-1718	Draft genome
27	2010-25654-F	Draft genome
28	2010-25654-O	Draft genome
29	2010-30795	Draft genome
30	2010-30800	Draft genome
31	2010-31374	Draft genome
32	2010-33561	Draft genome
33	2010-34330	Draft genome
34	2010-347972	Draft genome
35	2010-36743	Draft genome
36	2010-378007-F	Draft genome
37	2010-378007-O	Draft genome
38	2010-43100	Draft genome
39	2010-6073	Draft genome
40	2010-8194	Draft genome
41	2010-88823	Draft genome
42	2012-164712	Draft genome
43	2012-191940	Draft genome
44	2012-37302	Draft genome
45	2013-101463	Draft genome

46	2013-39845	Draft genome
47	2013-42088	Draft genome
48	2013-87946	Draft genome
49	AAUH-10HCce	Draft genome
50	AAUH-10HCdes	Draft genome
51	AAUH-10HCdes2	Draft genome
52	AAUH-10HCdes3	Draft genome
53	AAUH-10HCdes4	Draft genome
54	AAUH-10HCdes5	Draft genome
55	AAUH-10HCdes6	Draft genome
56	AAUH-10HCdes7	Draft genome
57	AAUH-10HCtra	Draft genome
58	AAUH-10UCf2	Draft genome
59	AAUH-10UCil-a	Draft genome
60	AAUH-11HCf	Draft genome
61	AAUH-11HCo-a	Draft genome
62	AAUH-11UCdes-a	Draft genome
63	AAUH-11UCo	Draft genome
64	AAUH-11UCsig-a	Draft genome
65	AAUH-12CDce	Draft genome
66	AAUH-12CDdes2	Draft genome
67	AAUH-12CDdes3	Draft genome
68	AAUH-12CDdes4	Draft genome
69	AAUH-12CDo	Draft genome
70	AAUH-12CDrec-a	Draft genome
71	AAUH-12CDsig	Draft genome
72	AAUH-12CDti2-a	Draft genome
73	AAUH-12CDti4-a	Draft genome
74	AAUH-12CDti5-a	Draft genome
75	AAUH-12CDtra2-a	Draft genome
76	AAUH-12CDtra-a	Draft genome
77	AAUH-12HCf	Draft genome
78	AAUH-14HCce	Draft genome
79	AAUH-15HCti	Draft genome
80	AAUH-15UCdp	Draft genome
81	AAUH-15UCdp-a	Draft genome
82	AAUH-15UCpp	Draft genome
83	AAUH-16UCdp	Draft genome
84	AAUH-16UCdp3	Draft genome
85	AAUH-16UCdp5	Draft genome
86	AAUH-16UCf	Draft genome
87	AAUH-16UCf2	Draft genome
88	AAUH-16UCf3	Draft genome
89	AAUH-16UCo-a	Draft genome
90	AAUH-19HCf	Draft genome
91	AAUH-19HCf2	Draft genome
92	AAUH-1Dasc	Draft genome
93	AAUH-1Dce-a	Draft genome
94	AAUH-1Dtra	Draft genome
95	AAUH-2010376221	Draft genome

96	AAUH-2012179281	Draft genome
97	AAUH-20HCasc	Draft genome
98	AAUH-20HCrec-a	Draft genome
99	AAUH-20HCsig-a	Draft genome
100	AAUH-20UCf	Draft genome
101	AAUH-20UCo	Draft genome
102	AAUH-22UCpp-a	Draft genome
103	AAUH-25Df	Draft genome
104	AAUH-25Df3	Draft genome
105	AAUH-2HCtra	Draft genome
106	AAUH-35UCdp	Draft genome
107	AAUH-35UCf	Draft genome
108	AAUH-35UCil2-a	Draft genome
109	AAUH-35UCil3-a	Draft genome
110	AAUH-35UCil4-a	Draft genome
111	AAUH-35UCil-a	Draft genome
112	AAUH-35UCpp	Draft genome
113	AAUH-37UCf	Draft genome
114	AAUH-37UCo-a	Draft genome
115	AAUH-39CDf	Draft genome
116	AAUH-39CDrec-a	Draft genome
117	AAUH-39CDti-a	Draft genome
118	AAUH-3HCce2	Draft genome
119	AAUH-3HCo	Draft genome
120	AAUH-3UCce	Draft genome
121	AAUH-3UCce2	Draft genome
122	AAUH-40UCf	Draft genome
123	AAUH-43UCce-a	Draft genome
124	AAUH-43UCf	Draft genome
125	AAUH-44UCsig6	Draft genome
126	AAUH-47UCil	Draft genome
127	AAUH-47UCil-a	Draft genome
128	AAUH-48UCdp-a	Draft genome
129	AAUH-48UCil-a	Draft genome
130	AAUH-48UCo-a	Draft genome
131	AAUH-49UCf	Draft genome
132	AAUH-49UCil-a	Draft genome
133	AAUH-49UCpp-a	Draft genome
134	AAUH-4UCti	Draft genome
135	AAUH-4UCti-a	Draft genome
136	AAUH-51UCf	Draft genome
137	AAUH-55UCtra-a	Draft genome
138	AAUH-58UCo	Draft genome
139	AAUH-59UCpp-a	Draft genome
140	AAUH-5CDo	Draft genome
141	AAUH-6HCo-a	Draft genome
142	AAUH-7UCil	Draft genome
143	AAUH-8HCo	Draft genome
144	AAUH-8HCo-a	Draft genome
145	AAUH-8UCo	Draft genome

146	AAUH-8UCpp	Draft genome
147	AAUH-8UCpp-a	Draft genome
148	AAUH-9HCasc	Draft genome
149	AAUH-9HCce	Draft genome
150	AAUH-9UCdp	Draft genome
151	AAUH-9UCpp	Draft genome
152	ATCC 33237	Fully sequenced
153	ATCC 51561	Draft genome
154	ATCC 51562	Draft genome
155	AUS22-Bd2	Draft genome
156	B124_Slimy-large	Draft genome
157	B124_Slimy-small	Draft genome
158	B124_Small-clear	Draft genome
159	B124_Small-grey	Draft genome
160	B38_Tiny-mucoid	Draft genome
161	CCUG 19995	Draft genome
162	H10O-S1	Draft genome
163	H11O-S1	Draft genome
164	H11O-S2	Draft genome
165	H12O-S1	Draft genome
166	H14O-S1	Draft genome
167	H15O-S1	Draft genome
168	H16O-S1	Draft genome
169	H17O-S1	Draft genome
170	H19O-S1	Draft genome
171	H1O1	Draft genome
172	H20O-S1	Draft genome
173	H21O-S1	Draft genome
174	H21O-S2	Draft genome
175	H21O-S3	Draft genome
176	H21O-S5	Draft genome
177	H22O-S1	Draft genome
178	H23O-S1	Draft genome
179	H24O-S1	Draft genome
180	H25O-S1	Draft genome
181	H26O-S1	Draft genome
182	H27O-S1	Draft genome
183	H28O-S1	Draft genome
184	H28O-S2	Draft genome
185	H29O-S1	Draft genome
186	H30O-S1	Draft genome
187	H3O1	Draft genome
188	H7O-S1	Draft genome
189	H9O-S1	Draft genome
190	H9O-S2	Draft genome
191	Lasto127.99	Draft genome
192	Lasto205.94	Draft genome
193	Lasto220.96	Draft genome
194	Lasto28.99	Draft genome
195	Lasto393.96	Draft genome

196	Lasto61.99	Draft genome
197	Lasto64.99	Draft genome
198	P10CDO-S1	Draft genome
199	P10CDO-S2	Draft genome
200	P11CDO-S1	Draft genome
201	P13UCO-S1	Draft genome
202	P13UCO-S3	Draft genome
203	P15UCO-S2	Draft genome
204	P16UCO-S2	Draft genome
205	P18CDO-S1	Draft genome
206	P19CDO-S1	Draft genome
207	P1CDO2	Draft genome
208	P1CDO3	Draft genome
209	P20CDO-S1	Draft genome
210	P20CDO-S2	Draft genome
211	P20CDO-S3	Draft genome
212	P20CDO-S4	Draft genome
213	P21CDO-S1	Draft genome
214	P21CDO-S2	Draft genome
215	P21CDO-S4	Draft genome
216	P24CDO-S2	Draft genome
217	P24CDO-S3	Draft genome
218	P24CDO-S4	Draft genome
219	P25CDO-S3	Draft genome
220	P26UCO-S1	Draft genome
221	P26UCO-S2	Draft genome
222	P27CDO-S1	Draft genome
223	P27CDO-S2	Draft genome
224	P28CDO-S1	Draft genome
225	P2CDO3	Draft genome
226	P2CDO4	Fully sequenced
227	P2CDO-S6	Draft genome
228	P3UCB1	Draft genome
229	P3UCO1	Draft genome
230	P6CDO1	Draft genome
231	P7UCO-S2	Draft genome
232	RCH 26	Draft genome
233	RMIT-JF1	Draft genome
234	UNSW1	Draft genome
235	UNSW2	Draft genome
236	UNSW3	Draft genome
237	UNSWCD	Draft genome
238	UNSWCS	Draft genome
239	MGYG-HGUT-01392	Draft genome
240	MGYG-HGUT-02425	Draft genome
241	MGYG-HGUT-02426	Draft genome
242	MGYG-HGUT-02427	Draft genome
243	MGYG-HGUT-02428	Draft genome
244	MGYG-HGUT-02430	Draft genome
245	MGYG-HGUT-02431	Draft genome

246	MGYG-HGUT-02432	Draft genome
247	MGYG-HGUT-02433	Draft genome
248	MGYG-HGUT-02434	Draft genome
249	MGYG-HGUT-02435	Draft genome
