# Supplementary material for: Analyses of energy metabolism and stress defence provide insights into Campylobacter concisus growth and pathogenicity
Source: Gut Pathog. 2020 Mar 5;12:13. doi: 10.1186/s13099-020-00349-6 (PMC7059363; doi:10.1186/s13099-020-00349-6)
Supplement: Supplementary file 11 — Additional file 11: Table S11. Genes encoding electron donors and acceptors investigated in C. concisus as referenced from C. jejuni subsp. jejuni NCTC 11168 and C. jejuni subsp. jejuni strain 81116. [file 13099_2020_349_MOESM11_ESM.pdf]

Analyses of energy metabolism and stress defence provide insights into *Campylobacter concisus* growth and pathogenicity

Table S11: Genes encoding electron donors and acceptors investigated in *C. concisus* as referenced from *C. jejuni* subsp. *jejuni* NCTC 11168 and *C. jejuni* subsp. *jejuni* strain 81116

Gene name	Locus tag	Function	Reference
oorA	*cj0536*	2-oxoglutarate:acceptor oxidoreductase, alpha subunit, involved in conversion of 2-oxoglutarate to succinyl coA	21
oorB	*cj0537*	2-oxoglutarate ferredoxin oxidoreductase, beta subunit, involved in conversion of 2-oxoglutarate to succinyl coA	21
oorC	*cj0538*	2-oxoglutarate oxidoreductase, gamma subunit, involved in conversion of 2-oxoglutarate to succinyl coA	21
oorD	*cj0535*	2-oxoglutarate:acceptor oxidoreductase, delta subunit, involved in conversion of 2-oxoglutarate to succinyl coA	21
nuoA	*cj1579c*	integral membrane protein subunit of NADH dehydrogenase/complex I	21
nuoB	*cj1578c*	peripheral membrane protein subunit of NADH dehydrogenase/complex I	21
nuoC	*cj1577c*	peripheral membrane protein subunit of NADH dehydrogenase/complex I	21
nuoD	*cj1576c*	peripheral membrane protein subunit of NADH dehydrogenase/complex I	21
-	*cj1575c*	NuoE paralog	21
-	*cj1574c*	NuoF paralog	21
nuoG	*cj1573c*	peripheral membrane protein subunit of NADH dehydrogenase/complex I	21
nuoH	*cj1572c*	integral membrane protein subunit of NADH dehydrogenase/complex I	21
nuoI	*cj1571c*	peripheral membrane protein subunit of NADH dehydrogenase/complex I	21
nuoJ	*cj1570c*	integral membrane protein subunit of NADH dehydrogenase/complex I	21
nuoK	*cj1569c*	integral membrane protein subunit of NADH dehydrogenase/complex I	21
nuoL	*cj1568c*	integral membrane protein subunit of NADH dehydrogenase/complex I	21
nuoM	*cj1567c*	integral membrane protein subunit of NADH dehydrogenase/complex I	21
nuoN	*cj1566c*	integral membrane protein subunit of NADH dehydrogenase/complex I	21
flavodoxin	*cj1382c*	electron transfer protein containing flavin mononucleotide (FMN)	21
fdhA	*cj1511c*	selenocysteine containing molybdoprotein equivalent to the *E. coli* 110 kDa FdnG subunit	22

fdhB	*cj1510c*	iron-sulphur subunit equivalent to the *E. coli* 32-kDa FdnH subunit.	22
fdhC	*cj1509c*	cytochrome b subunit equivalent to the *E. coli* 20-kDa FdnI subunit	22
fdhD	*cj1508c*	required for activity of the formate dehydrogenase enzyme complex	22
fumC	*cj1364c*	fumarate hydratase reversibly converts fumarate to malate	23
-	*cj0414*	gluconate 2-dehydrogenase gamma chain, involved in conversion of gluconate to 2-dehydro-D-gluconate	24
-	*cj0415*	gluconate 2-dehydrogenase alpha chain, involved in conversion of gluconate to 2-dehydro-D-gluconate	24
*edd**	*JJD26997_1271*	phosphogluconate dehydratase catalyzes the dehydration of 6-phospho-D-gluconate to 2-dehydro-3-deoxy-6-phospho-D-gluconate.	25
*eda**	*JJD26997_1272*	2-keto-3-deoxy-6-phosphogluconate aldolase (KDPG) catalyzes the reversible, stereospecific retro-aldol cleavage of KDPG to pyruvate and D-glyceraldehyde-3-phosphate	25
hydA	*cj1267c*	hydrogenase small subunit Fe–S protein	26
hydB	*cj1266c*	hydrogenase large subunit containing the NiFe active site responsible for accepting electrons from hydrogen	26
hydC	*cj1265c*	membrane-anchored b-type cytochrome subunit of hydrogenase	26
hydD	*cj1264c*	protease involved in enzyme maturation of hydrogenase	26
hypA	*cj0627*	involved in enzyme maturation of hydrogenase	23
hypB	*cj0623*	involved in enzyme maturation of hydrogenase	23
hypC	*cj0624*	involved in enzyme maturation of hydrogenase	23
hypD	*cj0625*	involved in enzyme maturation of hydrogenase	23
hypE	*cj0626*	involved in enzyme maturation of hydrogenase	23
hypF	*cj0622*	involved in enzyme maturation of hydrogenase	23
nikZ	*cj1484c*	nickel transporter	26
	cj1585	lactate dehydrogenase responsible for oxidation of lactate into pyruvate	27
lutA	*cj0073*	oxidoreductase subunit of lactate oxidase responsible for oxidation of lactate into pyruvate	27
lutB	*cj0074*	iron–sulphur subunit which transfers L-lactate-derived electrons to the respiratory chain via the iron–sulphur centres in LutB	27
lutC	*cj0075*	uncertain function	27

mdh	*cj0532*	malate dehydrogenase reversibly converts malate to oxaloacetate	23
por	*cj1476c*	pyruvate oxidoreductase	28
frdA	*cj0409*	fumarate reductase flavoprotein subunit involved in reversible reduction of fumarate	29
frdB	*cj0410*	fumarate reductase iron-sulphur subunit involved in reversible reduction of fumarate	29
frdC	*cj0408*	fumarate reductase cytochrome b subunit involved in reversible reduction of fumarate	29
sorA	*cj0004c*	monohaem cytochrome c oxidoreductase involved in sulphite oxidation to sulphide	30
sorB	*cj0005c*	molydopterin oxidoreductase involved in sulphite oxidation to sulphide	30
mfrA	*cj0437*	periplasmic methylmenaquinol:fumarate reductase, MfrA subunit involved in reduction of fumarate to succinate	31
mfrB	*cj0438*	periplasmic methylmenaquinol:fumarate reductase, MfrB subunit involved in reduction of fumarate to succinate	31
mfrE	*cj0439*	periplasmic methylmenaquinol:fumarate reductase, MfrE subunit involved in essential for correct transport of MfrA through the twin-arginine transport system.	31
napA	*cj0780*	catalytic subunit of nitrate reductase involved in reduction of nitrate to ammonia	32
napG	*cj0781*	electron transfer subunit of nitrate reductase involved in reduction of nitrate to nitrite	32
napH	*cj0782*	electron transfer subunit of nitrate reductase involved in reduction of nitrate to nitrite	32
napB	*cj0783*	electron transfer subunit of nitrate reductase involved in reduction of nitrate to nitrite	32
napL	*cj0784*	subunit of nitrate reductase with unknown function	32
napD	*cj0785*	‘Proof-reading’ chaperone required for export of NapA by twin-arginine transport system	32
nrfA	*cj1357c*	nitrite reductase catalytic subunit involved in reduction of nitrite to ammonia	20
nrfH	*cj1358c*	nitrite reductase electron transfer subunit involved in reduction of nitrite to ammonia	20
ccoN	*cj1490c*	cbb3-type cytochrome c oxidase subunit involved in reduction of oxygen to water	33
ccoO	*cj1489c*	cbb3-type cytochrome c oxidase subunit	33

		involved in reduction of oxygen to water	
ccoQ	*cj1488c*	cbb3-type cytochrome c oxidase subunit involved in reduction of oxygen to water	33
ccoP	*cj1487c*	cbb3-type cytochrome c oxidase subunit involved in reduction of oxygen to water	33
petA	*cj1186c*	cytochrome bc1 subunit	34
petB	*cj1185c*	cbb3-type cytochrome c oxidase subunit involved in reduction of oxygen to water	34
petC	*cj1184c*	cbb3-type cytochrome c oxidase subunit involved in reduction of oxygen to water	34
cioA	*cj0081*	cyanide-insensitive oxidase subunit involved in reduction of oxygen to water	33
cioB	*cj0082*	cyanide-insensitive oxidase subunit involved in reduction of oxygen to water	33
torA	*cj0264c*	molybdoenzyme that reduces trimethylamine N-oxide or dimethyl sulphoxide to trimethylamine/dimethyl sulphide	35
torC	*cj0265c*	monoheme c-type cytochrome involved in reducing trimethylamine N-oxide or dimethyl sulphoxide to trimethylamine/dimethyl sulphide	35
tsdA	*c8j_0815*	tetrathionate reductase reduces tetrathionate to thiosulphate	36
	c8j_0040	tetrathionate reductase reduces tetrathionate to thiosulphate	36
mqo	*cj0393c*	malate quinone oxidoreductase oxidises malate to oxaloacetate	37
