# Supplementary material for: Analyses of energy metabolism and stress defence provide insights into Campylobacter concisus growth and pathogenicity
Source: Gut Pathog. 2020 Mar 5;12:13. doi: 10.1186/s13099-020-00349-6 (PMC7059363; doi:10.1186/s13099-020-00349-6)
Supplement: Supplementary file 12 — Additional file 12. Additional references cited in additional files 7, 9 and 11. [file 13099_2020_349_MOESM12_ESM.docx]

Additional references which were cited in Additional file 7, 9 and 11.

1. Velayudhan, J., Jones, M. A., Barrow, P. A. & Kelly, D. J. L-serine catabolism via an oxygen-labile L-serine dehydratase is essential for colonization of the avian gut by *Campylobacter jejuni*. *Infection and immunity* 72, 260-268 (2004).
2. Hofreuter D, Mohr J, Wensel O, Rademacher S, Schreiber K, Schomburg D, et al. Contribution of amino acid catabolism to the tissue specific persistence of *Campylobacter jejuni* in a murine colonization model. PloS one. 2012;7(11):e50699-e.
3. Wösten, M. M. S. M., van de Lest, C. H. A., van Dijk, L. & van Putten, J. P. M. Function and Regulation of the C4-Dicarboxylate Transporters in *Campylobacter jejuni.* Frontiers in microbiology 8, 174-174 (2017).
4. Guccione E, Del Rocio Leon-Kempis M, Pearson BM, Hitchin E, Mulholland F, Van Diemen PM, et al. Amino acid-dependent growth of *Campylobacter jejuni:* key roles for aspartase (AspA) under microaerobic and oxygen-limited conditions and identification of AspB (*Cj0762*), essential for growth on glutamate. Molecular microbiology. 2008;69(1):77-93.
5. Hofreuter, D., Novik, V. & Galán, J. E. Metabolic Diversity in *Campylobacter jejuni* Enhances Specific Tissue Colonization. Cell Host & Microbe 4, 425-433 (2008).
6. Del Rocio Leon-Kempis, M., Guccione, E., Mulholland, F., Williamson, M. P. & Kelly, D. J. The *Campylobacter jejuni* PEB1a adhesin is an aspartate/glutamate-binding protein of an ABC transporter essential for microaerobic growth on dicarboxylic amino acids. Molecular microbiology 60, 1262-1275 (2006).
7. Lin, A. E. et al. Atypical Roles for *Campylobacter jejuni* Amino Acid ATP Binding Cassette Transporter Components PaqP and PaqQ in Bacterial Stress Tolerance and Pathogen-Host Cell Dynamics. Infection and immunity 77, 4912-4924 (2009).
8. Sakamoto, N., Kotre, A. M. & Savageau, M. A. Glutamate dehydrogenase from *Escherichia coli*: purification and properties. *Journal of bacteriology* 124, 775-783 (1975).
9. Ribardo, D. A. & Hendrixson, D. R. Analysis of the LIV system of *Campylobacter jejuni* reveals alternative roles for LivJ and LivK in commensalism beyond branched-chain amino acid transport. Journal of bacteriology 193, 6233-6243 (2011).
10. Bingham-Ramos, L. K. & Hendrixson, D. R. Characterization of Two Putative Cytochrome *c* Peroxidases of *Campylobacter jejuni* Involved in Promoting Commensal Colonization of Poultry. *Infection and immunity* 76, 1105-1114 (2008).
11. Oh, E. & Jeon, B. Role of Alkyl Hydroperoxide Reductase (AhpC) in the Biofilm Formation of *Campylobacter jejuni*. *PLOS ONE* 9, e87312 (2014).
12. Atack, J. M., Harvey, P., Jones, M. A. & Kelly, D. J. The *Campylobacter jejuni* Thiol Peroxidases Tpx and Bcp Both Contribute to Aerotolerance and Peroxide-Mediated Stress Resistance but Have Distinct Substrate Specificities. *Journal of Bacteriology* 190, 5279-5290 (2008).
13. Flint A, Sun Y-Q, Butcher J, Stahl M, Huang H, Stintzi A. Phenotypic Screening of a Targeted Mutant Library Reveals *Campylobacter jejuni* Defenses against Oxidative Stress. Infection and immunity. 2014;82(6):2266-75.
14. Ishikawa, T. *et al.* The iron-binding protein Dps confers hydrogen peroxide stress resistance to *Campylobacter jejuni*. *J Bacteriol* 185, 1010-1017, doi:10.1128/jb.185.3.1010-1017.2003 (2003).
15. Huergo, L. F., Rahman, H., Ibrahimovic, A., Day, C. J. & Korolik, V. *Campylobacter jejuni* Dps protein binds DNA in the presence of iron or hydrogen peroxide. J Bacteriol 195, 1970-1978 (2013).
16. Wang, G. & Maier, R. J. An NADPH quinone reductase of *Helicobacter pylori* plays an important role in oxidative stress resistance and host colonization. Infection and immunity 72, 1391-1396 (2004).
17. Atack, J. M. & Kelly, D. J. Contribution of the stereospecific methionine sulphoxide reductases MsrA and MsrB to oxidative and nitrosative stress resistance in the food-borne pathogen *Campylobacter jejuni.* Microbiology 154, 2219-2230 (2008).
18. Elvers, K. T., Wu, G., Gilberthorpe, N. J., Poole, R. K. & Park, S. F. Role of an Inducible Single-Domain Hemoglobin in Mediating Resistance to Nitric Oxide and Nitrosative Stress in *Campylobacter jejuni* and *Campylobacter coli.* Journal of Bacteriology 186, 5332-5341 (2004).
19. Wainwright, L. M., Elvers, K. T., Park, S. F. & Poole, R. K. A truncated haemoglobin implicated in oxygen metabolism by the microaerophilic food-borne pathogen *Campylobacter jejuni*. Microbiology 151, 4079-4091 (2005).
20. Pittman MS, Elvers KT, Lee L, Jones MA, Poole RK, Park SF, et al. Growth of *Campylobacter jejuni* on nitrate and nitrite: electron transport to NapA and NrfA via NrfH and distinct roles for NrfA and the globin Cgb in protection against nitrosative stress. Molecular microbiology. 2007;63(2):575-90.
21. Weerakoon DR, Olson JW. The *Campylobacter jejuni* NADH:Ubiquinone Oxidoreductase (Complex I) Utilizes Flavodoxin Rather than NADH. Journal of Bacteriology. 2008;190(3):915-25.
22. Hoffman PS, Goodman TG. Respiratory physiology and energy conservation efficiency of *Campylobacter jejuni*. Journal of bacteriology. 1982;150(1):319-26.
23. Parkhill J, Wren BW, Mungall K, Ketley JM, Churcher C, Basham D, et al. The genome sequence of the food-borne pathogen *Campylobacter jejuni* reveals hypervariable sequences. Nature. 2000;403(6770):665-8.
24. Kelly DJ. Complexity and Versatility in the Physiology and Metabolism of *Campylobacter jejuni*. Campylobacter , Third Edition: American Society of Microbiology; 2008.
25. Vegge CS, Jansen van Rensburg MJ, Rasmussen JJ, Maiden MCJ, Johnsen LG, Danielsen M, et al. Glucose Metabolism via the Entner-Doudoroff Pathway in *Campylobacter:* A Rare Trait that Enhances Survival and Promotes Biofilm Formation in Some Isolates. Frontiers in Microbiology. 2016;7(1877).
26. Howlett, R. M., Hughes, B. M., Hitchcock, A. & Kelly, D. J. Hydrogenase activity in the foodborne pathogen *Campylobacter jejuni* depends upon a novel ABC-type nickel transporter (NikZYXWV) and is SlyD-independent. Microbiology 158, 1645-1655 (2012).
27. Thomas MT, Shepherd M, Poole RK, van Vliet AHM, Kelly DJ, Pearson BM. Two respiratory enzyme systems in *Campylobacter jejuni NCTC 11168* contribute to growth on l-lactate. Environmental Microbiology. 2011;13(1):48-61.
28. Weerakoon DR, Borden NJ, Goodson CM, Grimes J, Olson JW. The role of respiratory donor enzymes in *Campylobacter jejuni* host colonization and physiology. Microbial Pathogenesis. 2009;47(1):8-15.
29. Weingarten RA, Taveirne ME, Olson JW. The dual-functioning fumarate reductase is the sole succinate:quinone reductase in *Campylobacter jejuni* and is required for full host colonization. J Bacteriol. 2009;191(16):5293-300.
30. Myers JD, Kelly DJ. A sulphite respiration system in the chemoheterotrophic human pathogen *Campylobacter jejuni*. Microbiology. 2005;151(1):233-42.
31. Guccione, E. et al. Reduction of fumarate, mesaconate and crotonate by Mfr, a novel oxygen-regulated periplasmic reductase in *Campylobacter jejuni.* Environmental Microbiology 12, 576-591 (2010).
32. Pittman MS, Kelly DJ. Electron transport through nitrate and nitrite reductases in *Campylobacter jejuni*. Biochem Soc Trans. 2005;33(Pt 1):190-2.
33. Jackson RJ, Elvers KT, Lee LJ, Gidley MD, Wainwright LM, Lightfoot J, et al. Oxygen Reactivity of Both Respiratory Oxidases in *Campylobacter jejuni*: the *cydAB* Genes Encode a Cyanide-Resistant, Low-Affinity Oxidase That Is Not of the Cytochrome *bd* Type. Journal of Bacteriology. 2007;189(5):1604-15.
34. Woodall CA, Jones MA, Barrow PA, Hinds J, Marsden GL, Kelly DJ, et al. *Campylobacter jejuni* gene expression in the chick cecum: evidence for adaptation to a low-oxygen environment. Infection and immunity. 2005;73(8):5278-85.
35. Sellars MJ, Hall SJ, Kelly DJ. Growth of *Campylobacter jejuni* supported by respiration of fumarate, nitrate, nitrite, trimethylamine-N-oxide, or dimethyl sulfoxide requires oxygen. Journal of bacteriology. 2002;184(15):4187-96.
36. Liu YW, Denkmann K, Kosciow K, Dahl C, Kelly DJ. Tetrathionate stimulated growth of *Campylobacter jejuni* identifies a new type of bi-functional tetrathionate reductase (TsdA) that is widely distributed in bacteria. Molecular microbiology. 2013;88(1):173-88.
37. Kather, B., Stingl, K., van der Rest, M. E., Altendorf, K. & Molenaar, D. Another unusual type of citric acid cycle enzyme in *Helicobacter pylori*: the malate:quinone oxidoreductase. J Bacteriol 182, 3204-3209 (2000).
